# Supplementary material for: Reliability of Whole-Exome Sequencing for Assessing Intratumor Genetic Heterogeneity
Source: Cell Rep. Author manuscript; Available in PMC 2018 Nov 28. (PMC6261536; doi:10.1016/j.celrep.2018.10.046)
Supplement: 1 [file NIHMS1512150-supplement-1.pdf]

Cell Reports, Volume *25*

Supplemental Information

Reliability of Whole-Exome Sequencing for Assessing Intratumor Genetic Heterogeneity

Weiwei Shi, Charlotte K.Y. Ng, Raymond S. Lim, Tingting Jiang, Sushant Kumar, Xiaotong Li, Vikram B. Wali, Salvatore Piscuoglio, Mark B. Gerstein, Anees B. Chagpar, Britta Weigelt, Lajos Pusztai, Jorge S. Reis-Filho, and Christos Hatzis

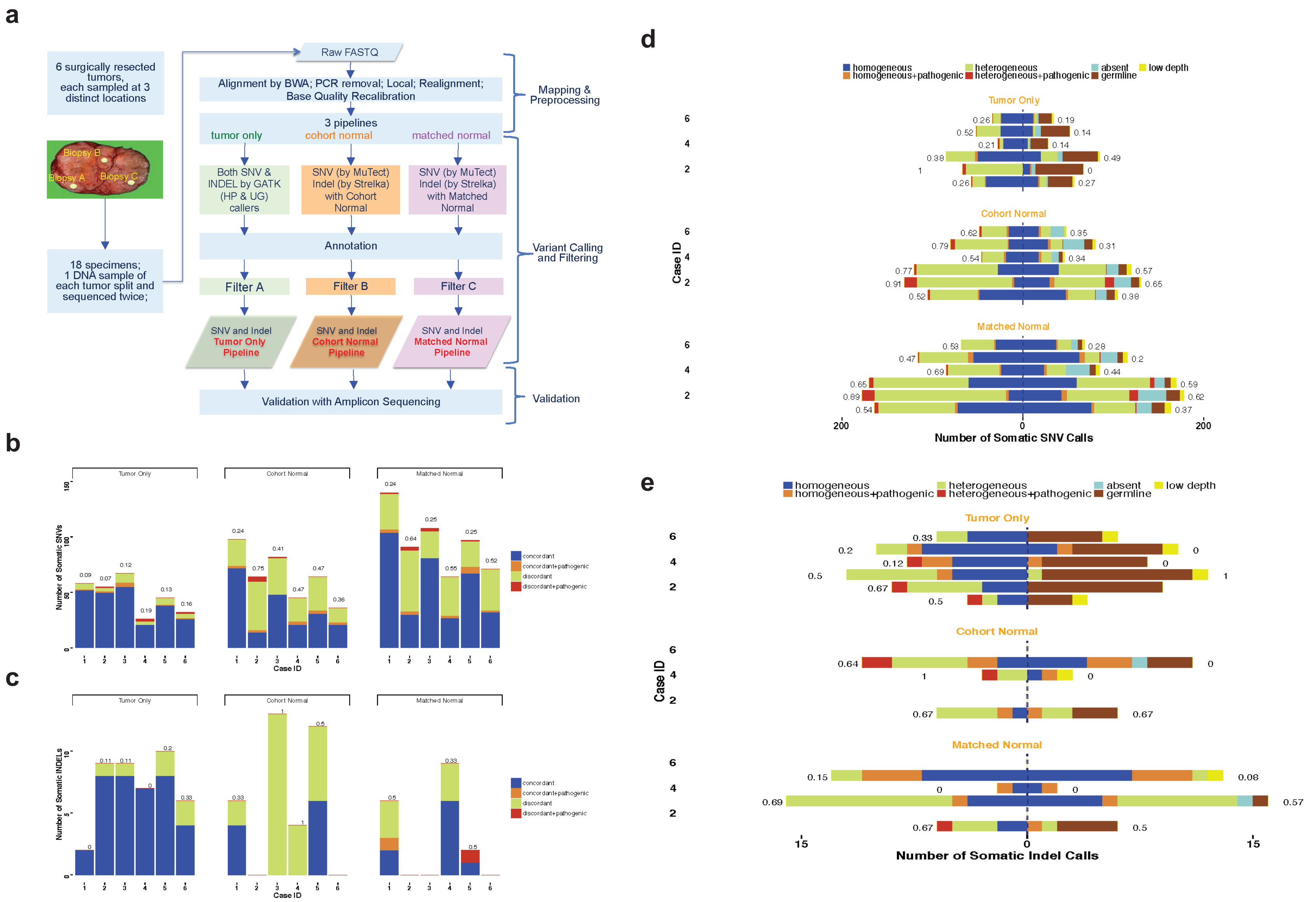
a
6 surgically resected tumors, each sampled at 3 distinct locations
Biopsy A
Biopsy B
Biopsy C
18 specimens; 1 DNA sample of each tumor split and sequenced twice;
Raw FASTQ
Alignment by BWA; PCR removal; Local; Realignment; Base Quality Recalibration
3 pipelines
tumor only
cohort normal
matched normal
Both SNV & INDEL by GATK (HP & UG) callers
SNV (by MuTect) Indel (by Strelka) with Cohort Normal
SNV (by MuTect) Indel (by Strelka) with Matched Normal
Annotation
Filter A
Filter B
Filter C
SNV and Indel Tumor Only Pipeline
SNV and Indel Cohort Normal Pipeline
SNV and Indel Matched Normal Pipeline
Validation with Amplicon Sequencing
Mapping & Preprocessing
Variant Calling and Filtering
Validation
b
Tumor Only
Cohort Normal
Matched Normal
Number of Somatic SNVs
Case ID
concordant
concordant+pathogenic
discordant
discordant+pathogenic
c
Number of Somatic INDELs
d
homogeneous
homogeneous+pathogenic
heterogeneous
heterogeneous+pathogenic
absent
germline
low depth
Case ID
Number of Somatic SNV Calls
e
Number of Somatic Indel Calls

Figure S1. (a) Experimental design and analytical pipelines for calling somatic variants, Related to Figure 1. DNA from three intra-tumor biopsies plus an additional technical replicate from each of six breast tumors was sequenced using whole exome sequencing. Raw DNA reads were processed and aligned to the hg19 or the GRCh37 human reference genome using BWA. Subsequently, somatic SNVs and INDELs were identified by three independent pipelines: 1) tumor-only using only the tumor DNA, 2) cohort-normal using the tumor DNA and a reference germline DNA pooled from ten unrelated blood DNA samples, and 3) matched-normal using the tumor DNA and blood DNA from the same patients. Each pipeline used different filters to exclude false positive somatic variants as described in the **Supplemental Experimental Procedures**. The identified somatic variants were subsequently validated by high-depth amplicon sequencing. Further details are provided in **Supplemental Experimental Procedures**.

(b-c) Comparison of technical variation in somatic variants identified from the three WES pipelines, Related to Figure 1; Figure 2. Comparison of the number of somatic **(b)** SNVs and **(c)** INDELs identified in the technical replicate pairs from each tumor using the tumor-only, cohort- normal, and matched-normal WES pipelines. Each bar corresponds to a technical replicate. The x-axis of these graphs is the total number of somatic variants identified by each pipeline in the pair of technical replicates from each tumor. The number above each bar is the Jaccard distance for the set of variants identified in each pair of technical replicates from a given tumor, which is a measure of the technical variation of the pipelines.

(d-e) Intratumor heterogeneity in somatic SNVs and INDELS detected by whole exome sequencing relative to deep sequencing, Related to Figure 1; Figure 2. Comparison of the number of somatic **(d)** SNVs and **(e)** INDELs identified in the intratumor biopsies from each tumor using the three WES pipelines (left) and their validation status according to high-depth amplicon sequencing (right). All putative WES somatic variants in the intratumor biopsies were validated by high-depth amplicon sequencing and classified as low depth (<50x), absent (VAF<1%), germline (tumor VAF<5x germline VAF), somatic and somatic (low VAF, **Table S3**). Homogeneous somatic variants were those identified in all three intratumor biopsies and heterogeneous were those detected in one or two biopsies. Pathogenicity of variants was assessed as described in **Supplemental Experimental Procedures**. The extent of intratumor heterogeneity in the somatic variant calls was quantified as the Jaccard distance for each case and is shown next to each bar.

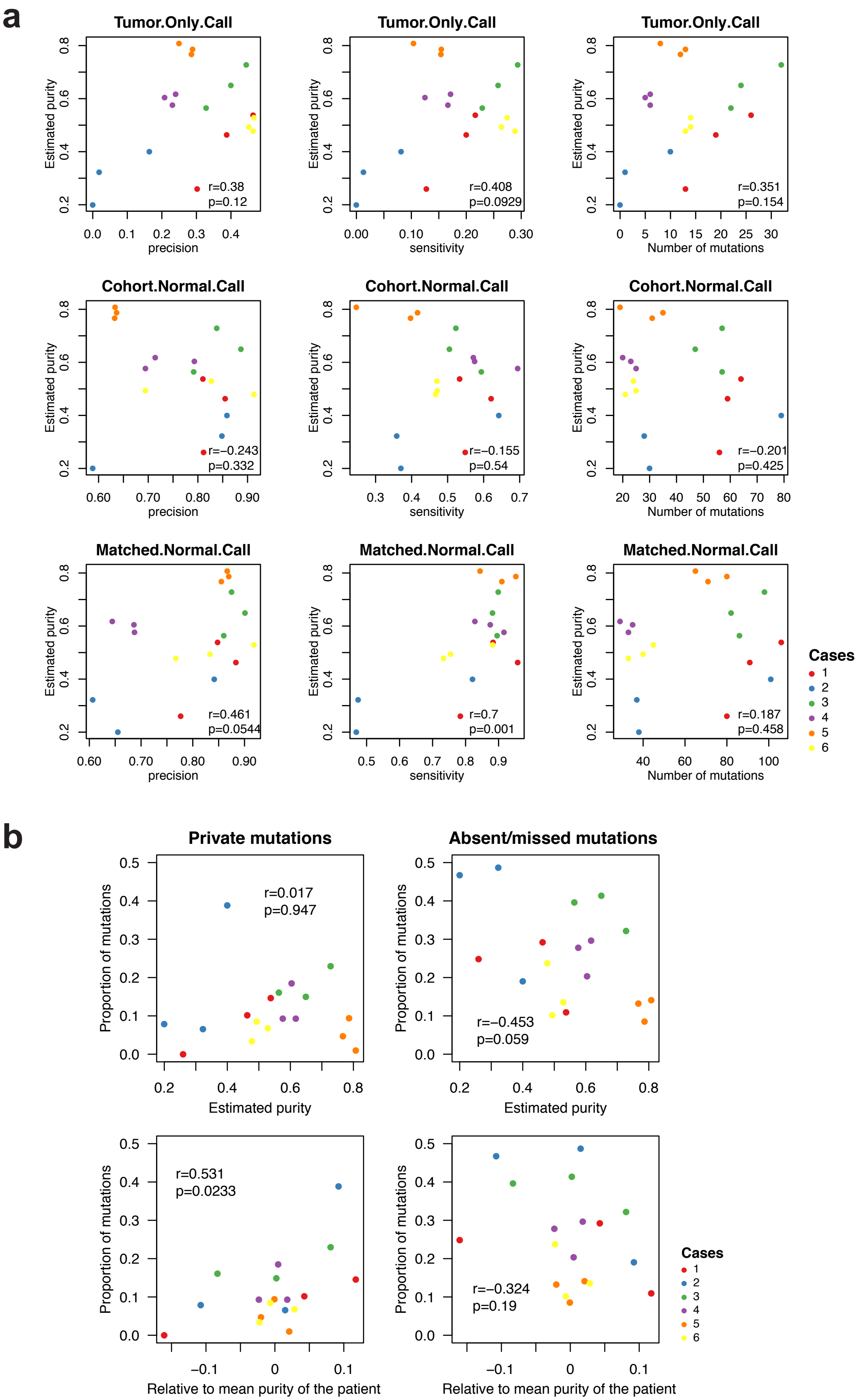
a
Tumor.Only.Call
Estimated purity
precision
r=0.38
p=0.12
Tumor.Only.Call
sensitivity
r=0.408
p=0.0929
Tumor.Only.Call
Number of mutations
r=0.351
p=0.154
Cohort.Normal.Call
precision
r=−0.243
p=0.332
Cohort.Normal.Call
sensitivity
r=−0.155
p=0.54
Cohort.Normal.Call
Number of mutations
r=−0.201
p=0.425
Matched.Normal.Call
precision
r=0.461
p=0.0544
Matched.Normal.Call
sensitivity
r=0.7
p=0.001
Matched.Normal.Call
Number of mutations
r=0.187
p=0.458
Cases
1
2
3
4
5
6
b
Private mutations
Proportion of mutations
Estimated purity
r=0.017
p=0.947
Absent/missed mutations
r=−0.453
p=0.059
r=0.531
p=0.0233
Relative to mean purity of the patient
r=−0.324
p=0.19
Cases
1
2
3
4
5
6

Figure S2. (a) Relationship between tumor purity and performance characteristics of the three WES analysis pipelines, Related to Figure 2. Precision and sensitivity are defined as in Fig. 2c. Each dot represents a tumor biopsy, color coded by the patients. Tumor purity was estimated using FACETS. Correlation was computed as Pearson correlation (r). The estimated purity for Case 2 biorep C, for which purity could not be estimated by FACETS, was set to 0.2.

(b) Relationship between tumor purity and intratumor genetic heterogeneity, Related to Figure 2. The proportion of private mutations (i.e. mutations found only in a given biopsy as a proportion of the union of all validated somatic mutations in a given patient, left) and absent/missed mutations (i.e. mutations found in at least one other biopsy but not the biopsy of interest) are plotted against estimated purity as defined by FACETS (top) and estimated purity relative to the mean purity of all intratumor biopsies of a given patient (bottom). Each dot represents a tumor biopsy, color coded by patients. Correlation was computed as Pearson correlation (r). The estimated purity for Case 2 biorep C, for which purity could not be estimated by FACETS, was set to 0.2.

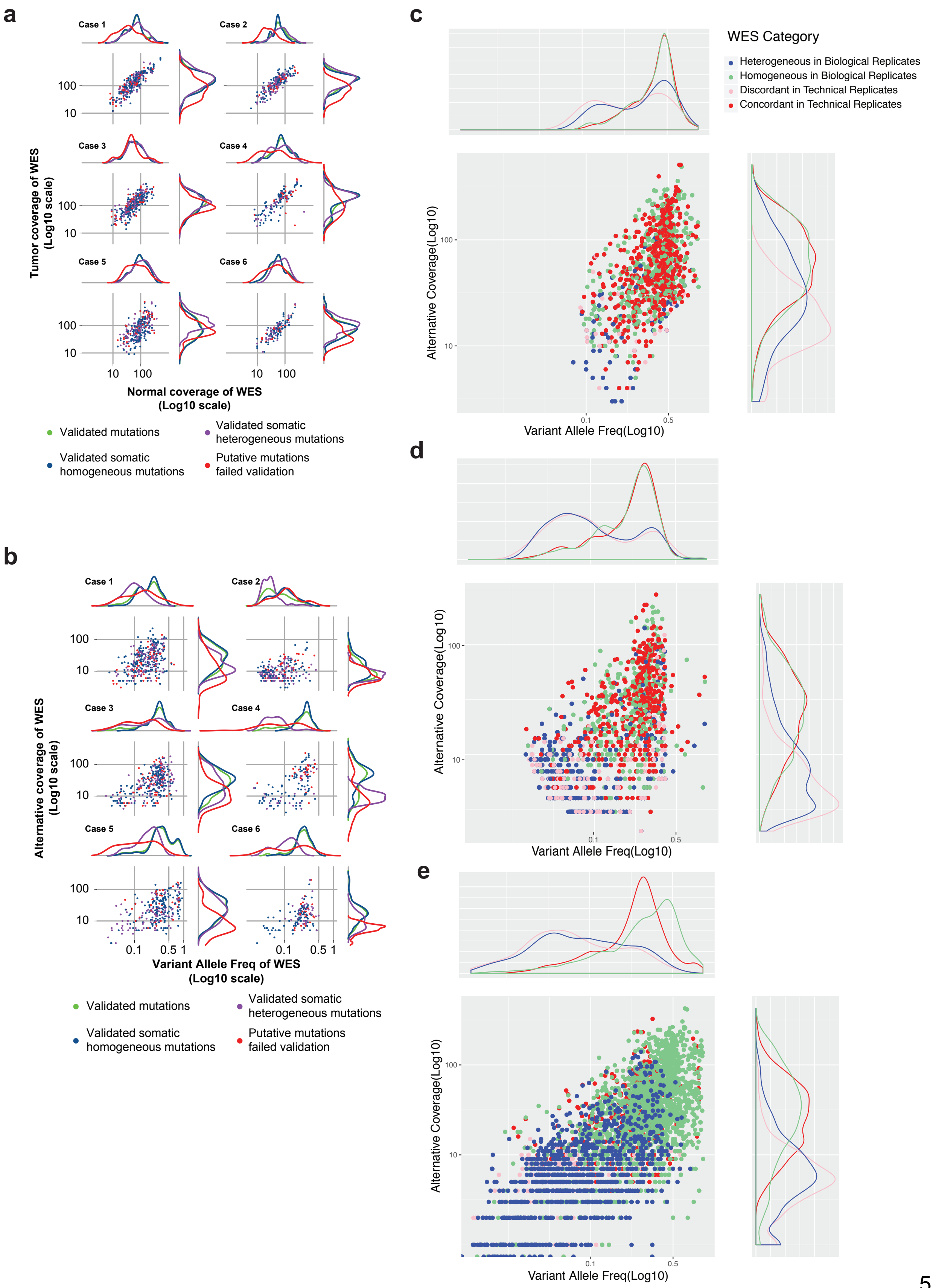
a
Case 1
Case 2
Case 3
Case 4
Case 5
Case 6
Tumor coverage of WES (Log10 scale)
Normal coverage of WES (Log10 scale)
Validated mutations
Validated somatic heterogeneous mutations
Validated somatic homogeneous mutations
Putative mutations failed validation
b
Alternative coverage of WES (Log10 scale)
Variant Allele Freq of WES (Log10 scale)
c
WES Category
Heterogeneous in Biological Replicates
Homogeneous in Biological Replicates
Discordant in Technical Replicates
Concordant in Technical Replicates
Alternative Coverage(Log10)
Variant Allele Freq(Log10)
d
e

Figure S3. (a) Coverage characteristics of true somatic variants and false positive mutations in the WES data for individual patients, using the matched-normal WES pipeline, Related to Figure 3. Total coverage in the tumor is plotted against the coverage in the matched normal sample of somatic mutations identified in all the specimens of a given patient. The validation status categories are the same as in panels Figure 3. Density kernel plots of the marginal distributions are included above and to the right of the scatter plots for each of the four categories of mutations.

(b) Coverage characteristics of true somatic variants and false positive mutations in the WES data for individual patients, using the matched-normal WES pipeline, Related to Figure 3. WES alternative allele coverage is plotted against VAF of somatic mutations identified in all the specimens of a given patient. The validation status categories are the same as in panels Figure 3. Density kernel plots of the marginal distributions are included above and to the right of the scatter plots for each of the four categories of mutations.

(c-e) Characteristics of variants called by WES pipelines in the intratumor biopsies, Related to Figure 3. Alternative allele coverage from WES versus variant allele fraction (log10 scale) for variants identified by **(c)** tumor-only, **(d)** cohort-normal or **(e)** matched-normal WES analysis pipelines in the intratumor biopsies from all cases. These calls include all putative somatic variants as defined by each of the pipelines prior to validation by amplicon sequencing. Variants are labeled as concordant in the technical replicates, discordant in the technical replicates, homogeneous if they were identified in all three biopsies, and heterogeneous if they were identified in one or two of the biopsies.

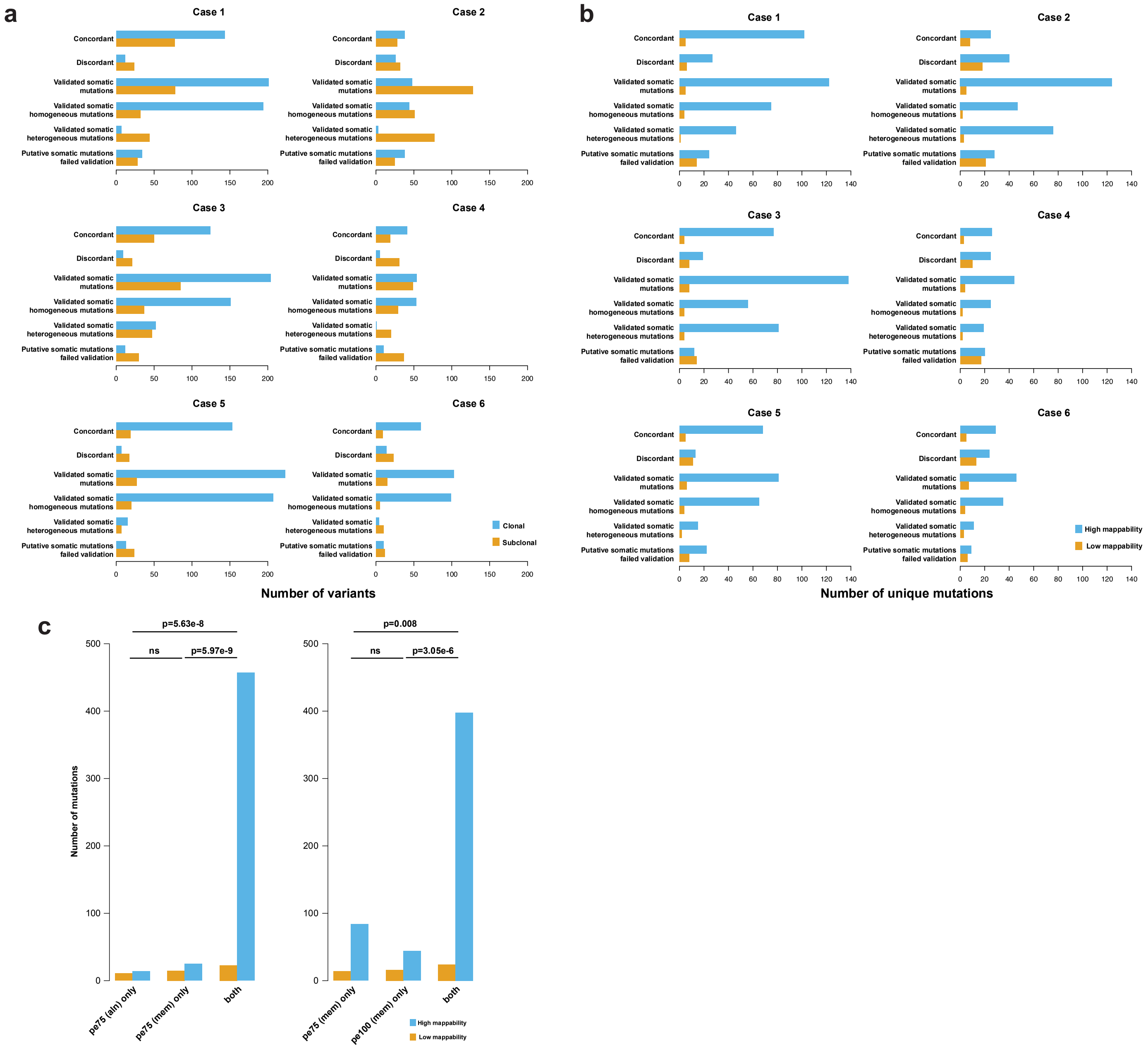
a
Case 1
Case 2
Case 3
Case 4
Case 5
Case 6
Concordant
Discordant
Validated somatic mutations
Validated somatic homogeneous mutations
Validated somatic heterogeneous mutations
Putative somatic mutations failed validation
Clonal
Subclonal
Number of variants
b
High mappability
Low mappability
Number of unique mutations
c
Number of mutations
p=5.63e-8
ns
p=5.97e-9
p=0.008
p=3.05e-6
pe75 (aln) only
pe75 (mem) only
both
pe100 (mem) only

Figure S4. (a) Clonality as defined by ABSOLUTE of true and artifactual somatic variants identified by the matched-normal WES pipeline for individual patients, Related to Figure 4. In each panel, the first two sets of bars enumerate the putative somatic variants identified as concordant or discordant in the technical replicates, whereas the bottom four sets of bars enumerate the somatic variants identified in intratumor biopsies and subsequently validated by high-depth amplicon sequencing.

(b) Mappability of true and artifactual somatic variants identified by the matched-normal WES pipeline for individual patients, Related to Figure 4. In each panel, the first two sets of bars enumerate the putative somatic variants identified as concordant or discordant in the technical replicates, whereas the bottom four sets of bars enumerate the somatic variants identified in intratumor biopsies and subsequently validated by high-depth amplicon sequencing. High mappability regions are regions with mappability score of 1.

(c) Mappability of somatic variants identified by the matched-normal WES pipeline, comparing the effects of alignment algorithms and read length, using 10 breast cancer cases from TCGA, Related to Figure 4. Comparison of mappability of somatic variants identified (left) between paired-end 75bp reads aligned with BWA aln and BWA mem and (right) between paired-end 75bp reads and paired-end 100bp reads aligned with BWA mem. Comparisons were performed using WES data from 10 tumor-normal pairs from the TCGA breast cancer cohort, originally sequenced using 100bp paired-end sequencing. To obtain 75bp reads, reads were trimmed to 75bp prior to alignment. For the 100bp reads, reads were aligned as is, then downsampled to 75% to match the overall sequencing depth of the trimmed 75bp reads. Somatic mutations were called using the matched-normal WES pipeline. Statistical comparisons were performed using Fisher exact tests.

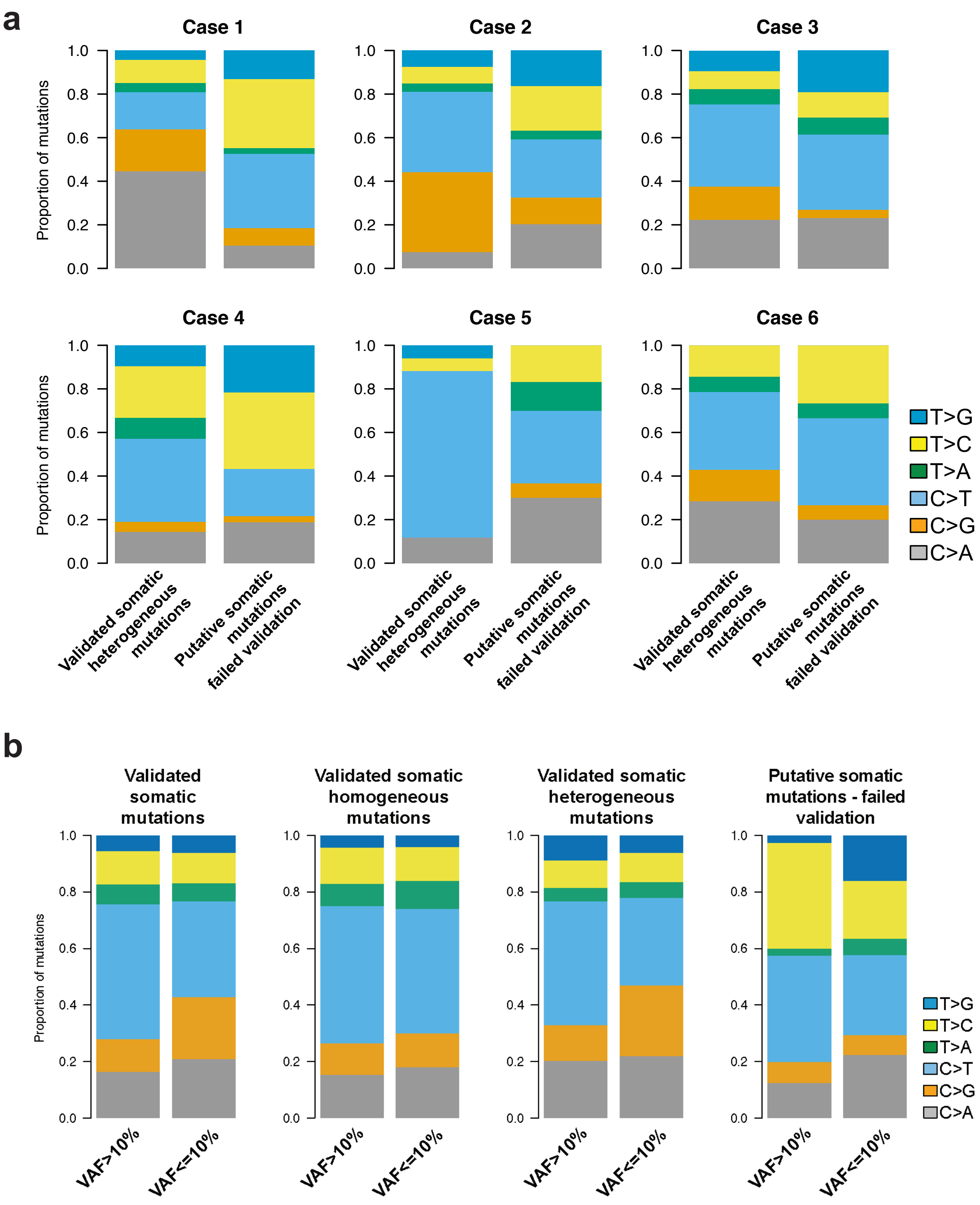
a
Case 1
Case 2
Case 3
Case 4
Case 5
Case 6
Proportion of mutations
0.0
0.2
0.4
0.6
0.8
1.0
Validated somatic heterogeneous mutations
Putative somatic mutations failed validation
T>G
T>C
T>A
C>T
C>G
C>A
b
Validated somatic mutations
Validated somatic homogeneous mutations
Validated somatic heterogeneous mutations
Putative somatic mutations - failed validation
VAF>10%
VAF<=10%

Figure S5. (a) Mutational spectra of true and artifactual somatic variants identified by the matched-normal WES pipeline for individual patients, Related to Figure 4. Comparison of the mutational spectra of validated somatic heterogenous mutations and artifactual somatic mutations that failed validation in all samples for individual patients. The reference base listed (C or T) includes the corresponding reverse complement (G or A).

(b) Mutational spectra of true and artifactual somatic variants identified by the matched-normal WES pipeline stratified by VAF, Related to Figure 4. Comparison of the mutational spectra of validated somatic, validated somatic homogeneous, validated somatic heterogenous and artifactual somatic mutations that failed validation in all samples. Mutations are stratified into VAF>10% and VAF<=10%. The reference base listed (C or T) includes the corresponding reverse complement (G or A).

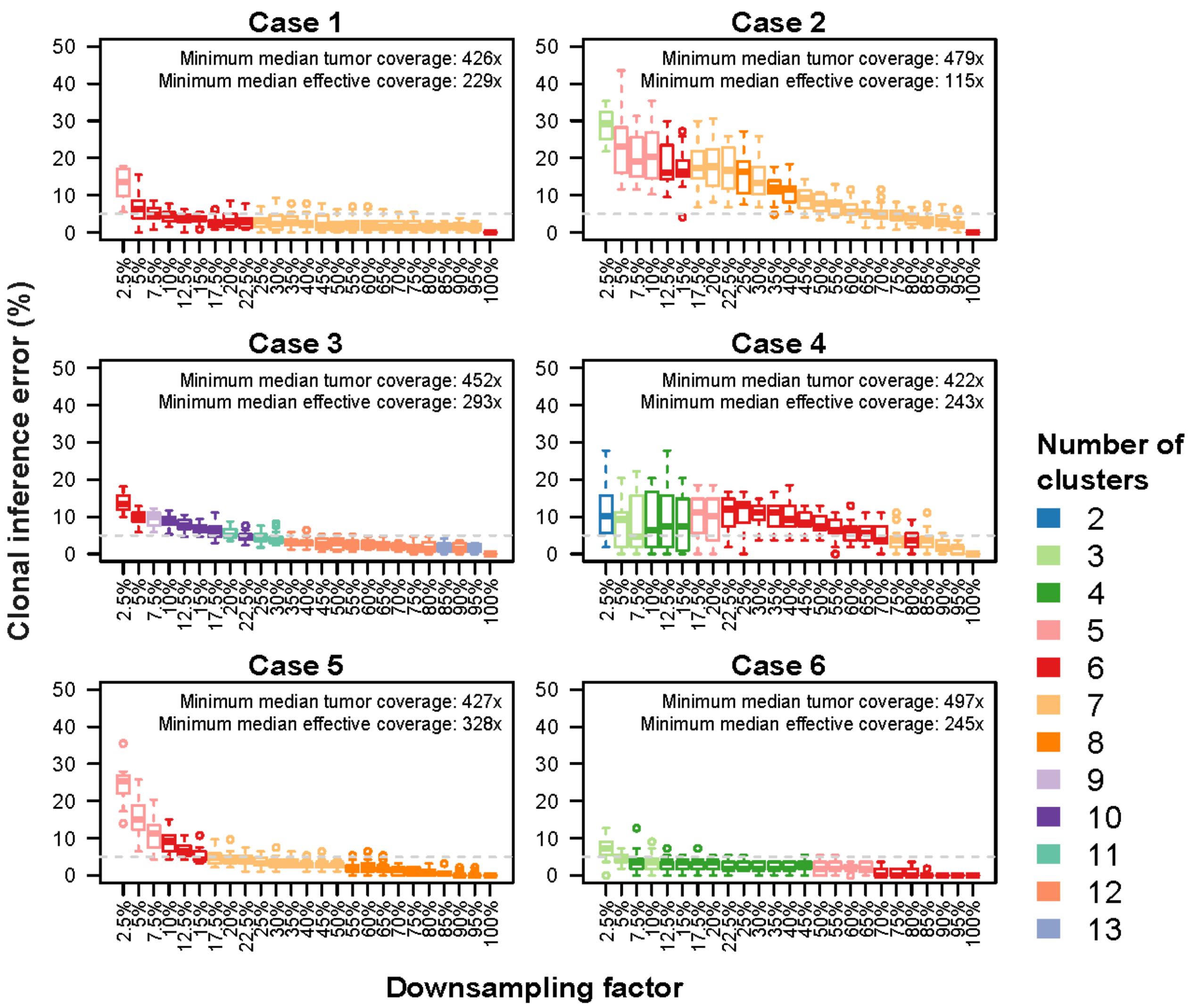
Case 1
Minimum median tumor coverage: 426x
Minimum median effective coverage: 229x
Case 2
Minimum median tumor coverage: 479x
Minimum median effective coverage: 115x
Case 3
Minimum median tumor coverage: 452x
Minimum median effective coverage: 293x
Case 4
Minimum median tumor coverage: 422x
Minimum median effective coverage: 243x
Case 5
Minimum median tumor coverage: 427x
Minimum median effective coverage: 328x
Case 6
Minimum median tumor coverage: 497x
Minimum median effective coverage: 245x
Clonal inference error (%)
Downsampling factor
Number of clusters
2
3
4
5
6
7
8
9
10
11
12
13

Figure S6. Impact of sequencing depth on clonal inference, Related to Figure 4. Boxplot showing clonal inference error at various degrees of downsampling sequencing depth. Clonal inference was performed using PyClone and clonal inference error was calculated in relation to no downsampling (i.e. 100% in the figure). Clonal inference error was calculated as 1-(the number of mutations in the same clusters as no downsampling/the total number of mutations). Downsampling was performed at 2.5% increments up to 25%, then at 5% increments, for 20 iterations. All somatic mutations were included, regardless if they would have been considered somatic at the reduced depth. Boxplots are colored according to the median number of clusters identified in the 20 runs. The minimum median tumor coverage refers to the minimum sample-level median depth of the mutations that were somatic in at least one sample in a given case (i.e. homogeneous or heterogeneous mutations). The median effective coverage refers to the sample-level median depth (as above) multiplied by its estimated tumor purity. Case 2 biorep C, for which purity could not be estimated by FACETS, was set to 0.2. The grey dotted line indicates 5% clustering error. Note the overlap between the minimum median tumor coverage and the minimum median effective coverage between the 4 cases with stable clonal inference (Cases 1, 3, 5, and 6) vs the 2 cases with unstable clonal inference (Cases 2 and 4).

SUPPLEMENTAL EXPERIMENTAL PROCEDURES

Tumor sample collection

Breast cancer samples were collected in the context of a prospective study to assess within-tumor genomic heterogeneity as previously published (Qi et al., 2015). Patients with newly diagnosed invasive breast cancer with tumor size > 2 cm were eligible and were recruited between 9th of January 2012 and 13th of November 2013 at the Yale Cancer Center. Tumor tissues were obtained with 2-3 punch biopsies at least 1 cm apart from 3 different regions of a tumor after pathologic gross examination had been completed. One biopsy from each location was formalin fixed and embedded in paraffin and the remaining biopsies were collected into RNAlater™ and stored at -80°C until DNA extraction. Tumor cellularity of each biopsy was assessed on the hematoxylin-and-eosin stained formalin-fixed sister biopsy from a given tumor location. We processed DNA for WES only if the biopsy cellularity was ≥50%. This study was approved by the Yale Cancer Center human investigations committee and all patients signed informed consent. Six tumors from this cohort with high quality DNA from all three biopsies and matched blood DNA were selected in this study to represent different breast cancer subtypes and disease spectra (**Table 1**).

Whole-exome sequencing

DNA from the three biopsies and matched blood from each of the six cases was extracted using the AllPrep Universal Kit (Qiagen). 1 µg genomic DNA was sheared to mean fragment length of 140 bp using the Covaris E210 instrument and purified by Magnetic AMPure XP beads (Beckman Coulter), and subsequently labeled with 6 base barcode during PCR amplification. The NimbleGen SeqCap EZ Human Exome Kit v2.0 (Roche) was used for exome capture following manufacturer's instructions. Intratumor biopsies and technical replicates were assigned uninformative identifiers to allow blinded sample processing, and subsequent library preparation and sequencing. Libraries were sequenced on Illumina HiSeq 2000 in paired-end 75-cycles mode at the Yale Center for Genome Analysis to a median depth of coverage of tumor samples and normal samples of 184 (range 92–211) and 90 (range 80–138), respectively **(Table S2**).

Whole-exome sequencing (WES) analysis pipelines

We used three different analytical pipelines for WES analysis (**Fig. S1a**). The single-sample "tumor-only" pipeline used only the reads derived from the tumor samples to define likely somatic mutations. Sequence reads were aligned to the human genome reference sequence version hg19 using the Burrows-Wheeler Aligner (Li and Durbin, 2009) (BWA, v0.6.2). PCR duplicates were removed using MarkDuplicates algorithm from Picard (version 1.47, http://picard.sourceforge.net/). Local realignment was performed using GATK (v3.1-1) (McKenna et al., 2010) around novel and known variant sites followed by GATK base quality recalibration. The overlap between the GATK HaplotypeCaller and UnifiedGenotyper algorithms was used to define mutations. Those identified by both callers were annotated by ANNOVAR (Yang and Wang, 2015) and non-exonic variants in regions with low mappability (Zook et al., 2014) or those outside the exome capture regions were excluded. We further excluded as putative germline SNPs those present in 1000Genomes phase1 (2014 Oct. http://www.1000genomes.org/), ESP6500 (http://evs.gs.washington.edu/EVS/), ExAC01 (http://exac.broadinstitute.org/about) or dbSNP (Build 138; variants not flagged as somatic or clinical or as having a minor allele frequency >1%).

The cohort-normal pipeline used an in-house normal reference obtained from 10 unrelated normal blood DNA samples sequenced using the same protocol, each downsampled to 20% and then pooled. Sequence reads were aligned to the human reference genome (GRCh37) using the Burrows-Wheeler Aligner (BWA, v0.6.2), followed by PCR duplicate removal, local realignment and base quality recalibration as described above. Somatic SNVs and small somatic insertions and deletions (INDELs) were defined by MuTect (Cibulskis et al., 2013) (v.1.1.4) and Strelka (Saunders et al., 2012) (v.1.0.14), respectively, using the pool of normal reads as reference and annotated by ANNOVAR (Yang and Wang, 2015). Recurrent variants with five or more occurrences in COSMIC (v.64) or ClinVar (http://www.ncbi.nlm.nih.gov/clinvar/) were whitelisted. Variants outside the target region were excluded. Low confidence somatic calls, defined as those with total coverage of fewer than 15 reads in the tumor, were present in at least five normal breast samples from the TCGA cohort (Koboldt et al., 2012a), considered likely germline in dbSNP (Build 138), or were present in ESP6500, 1000Genomes or ExAC01 were also excluded. We further removed variants with tumor variant allele fraction (VAF) < 5 times of that in the pooled normal DNA or with tumor VAF between 0.45 and 0.55. All remaining variants were manually inspected in the Integrative Genomics Viewer (Robinson et al.,

2011).

The "matched-normal" pipeline is considered the best-practice pipeline for identifying somatic events. Sequence alignment and processing up to base quality recalibration are the same as for the cohort-normal pipeline. Somatic SNVs and INDELs were detected using the sequencing reads derived from matched normal DNA from each patient as the reference. Somatic SNVs were defined using MuTect (Cibulskis et al., 2013); small INDELs were identified by the intersection of Strelka (Saunders et al., 2012) and VarScan 2 (Koboldt et al., 2012b), and further curated by manual inspection. Only variants at positions with total read depth >5 in both the tumor and normal were considered. Variants outside the target region or those supported by <5 reads were disregarded (De Mattos-Arruda et al., 2014; Martelotto et al., 2015). Variants covered by <20 reads in the germline of which more than one supported the mutant allele were disregarded. Variants covered by at least 20 reads in the germline were disregarded if the VAF in the tumor was < 5 times than that of the VAF in the germline. Variants present at global minor allele frequency >1% in dbSNP (version 138) were disregarded. Variants were annotated using SnpEff (Cingolani et al, 2012).

The identification of allele-specific copy number alterations (CNAs) and the estimation of tumor cellularity were performed using FACETS (Shen and Seshan, 2016), which performs a joint segmentation of the total and allelic copy ratio and infers allele-specific copy number states, using the reads derived from tumor samples and their matched normal counterparts. Regions of loss of heterozygosity were defined as regions having lesser (minor) copy number of zero.

Assessing technical variance of tumor cellularity estimated from WES

Estimates of tumor cellularity from technical replicate biopsies were obtained from FACETS (Shen and Seshan, 2016). We used a linear mixed-effects model with fixed and random intercept terms to estimate the mean cellularity and intratumor standard deviation. The error term provided an estimate of the within tumor or technical standard deviation of estimated cellularity.

Validation of putative somatic variants with high-depth amplicon sequencing (Ampliseq)

Variants identified by WES in both the biological and the technical replicates were subjected to validation with high-depth amplicon sequencing using custom Ampliseq panels on the same DNA on which WES was performed for all tumor and matched normal DNA from all six cases. Validation of putative WES somatic variants identified in the intratumor biopsies and technical replicates was performed separately. The validation panel for the intratumor biopsies included all putative mutations identified from at least one of the three pipelines (tumor-only, cohort-normal and matched-normal) described above. The validation panel for the technical replicates included putative mutations identified by the matched-normal pipeline only. Amplicons were successfully designed for 93.0% (1401/1508, range 91.0%–95.1% per patient) and 93.4% (741/793, range 85.5%–96.9% per patient) of the unique mutations for the biological replicates and the technical replicates, respectively. Putative mutations identified from WES for which amplicons could not be designed were excluded from further analyses.

Amplicon sequencing was performed to a median depth of 604x (range 363x–1519x) and 602x (range 460x–3102x) for the intratumor biopsies and technical replicates respectively (**Table S1**). Paired-end reads in FASTQ format were aligned to the reference human genome GRCh37 using the Torrent Mapping Alignment Program (TMAP, v3.4.1, https://github.com/iontorrent/TS/tree/master/Analysis/TMAP). Local realignment was performed using GATK (v3.1.1). Putative mutations were interrogated using pileup files generated using samtools mpileup (version 1.2 htslib 1.2.1)(Li, 2011), using reads with mapping quality of at least 1. For a given sample, mutations sequenced to ≤ 50x total depth were considered "low depth", and mutations present at VAF ≤ 1% were considered "absent". Mutations were considered "germline-like" if the VAF in the tumor was < 5 times than that of the VAF in the germline. The remaining mutations were considered validated to be "somatic". These definitions are summarized in **Table S2**.

For each pair of technical replicates, variants validated to be somatic in both samples were considered "concordant". Variants classified as low depth in either of the tumor samples were considered "low depth". Variants that were validated to be absent in both samples were considered "absent". Variants classified as germline-like in both tumor samples, or classified as germline-like in one sample and absent in the other sample, were considered "germline-like. Variants validated to be somatic in one of the two samples and germline or absent in the other sample were considered to be "discordant". Similarly, for the multiple biopsies from the same cancer, variants validated as somatic in all three biopsies were considered "homogeneous". Variants that were validated to be absent from all three biopsies were considered as "absent". Variants classified as low depth in any of the three biopsies were considered as "low depth". Variants that were validated to be germline-like in one of the biopsies and germline-like or absent in the other

two were considered "germline-like". The remaining variants that were validated as somatic in 1 or 2 of the biopsies and germline or absent in the other biopsies were considered as "heterogeneous". These definitions are summarized in **Table S2**.

Assessing technical variance and intra-tumor genetic heterogeneity (ITGH)

Concordance in putative somatic mutations as defined in the technical replicates by each of the three WES pipelines prior to validation was assessed using the Jaccard index (Levandowsky et al., 1971). The extent of discordance or technical noise involved in calling somatic mutations by a given pipeline was assessed by the Jaccard distance, which was defined as 1–Jaccard index, where 0 would indicate identical calls and 1 completely non-overlapping calls within each pair.

ITGH was assessed by comparing the somatic mutations identified in the three intratumor biopsies from the same tumor. The extent of ITGH for each case was then estimated by the Jaccard distance considering calls made on all three intratumor biopsies (1-homogeneous variants/union of somatic variants).

Identification of pathogenic and potentially pathogenic mutations

All validated non-synonymous mutations were classified as likely pathogenic or passenger mutations using *in silico* methods. For missense SNVs, their potential functional effects were assessed using a combination of MutationTaster (Schwarz et al., 2010) and CHASM (breast classifier) (Carter et al., 2009). SNVs defined as non-deleterious/passenger by both MutationTaster and CHASM were considered passenger mutations, as this combination was previously shown to have the highest negative predictive value (Martelotto et al., 2014). Non-passenger missense SNVs were defined as likely pathogenic if they were recurrent hotspot mutations (Chang et al., 2016), or predicted as "driver" or "cancer" alterations by CHASM or FATHMM (Shihab et al., 2013), respectively.

Pathogenicity of somatic in-frame indels was assessed using MutationTaster (Schwarz et al., 2010) and Protein Variation Effect Analyzer (PROVEAN) (Choi et al., 2012). In-frame indels predicted to be neutral by either were considered as passenger mutations, otherwise in-frame indels were considered likely pathogenic if they were associated with haploinsufficient affected genes (Dang et al., 2008), loss of the wild-type allele (based on FACETS (Shen and Seshan, 2016), see above), or at least one of the three cancer gene datasets (127 significant mutated genes (Kandoth et al., 2013a), the Cancer Gene Census (Futreal et al., 2004a) and Cancer5000-S gene set (Lawrence et al., 2014a)). Frameshift indels, splice donor/acceptor mutations and truncating mutations were considered potentially pathogenic if they were associated with loss of the wild-type allele (based on FACETS (Shen and Seshan, 2016)) or haploinsufficient affected genes (Dang et al., 2008), or affected cancer genes (Futreal et al., 2004b; Kandoth et al., 2013b; Lawrence et al., 2014b). Mutations that did not satisfy the above criteria were considered passenger mutations. Lists and characteristics of somatic variants detected by WES and Ampliseq on technical replicates are listed in **Table S3**. Somatic variants detected by WES and Ampliseq on the intratumor biopsies are listed in **Table S4**.

Identification of subclonal mutations

The clonality of putative somatic mutations identified from WES analysis was inferred using ABSOLUTE (Carter et al., 2012) using the segmented copy number log ratio from FACETS and the number of reads supporting the reference and the alternate alleles of the mutations obtained from WES as previously described (Ng et al., 2017a). The clonality of validated somatic mutations by Ampliseq was inferred using the number of reads supporting the reference and the alternate alleles of the mutations obtained by Ampliseq. A mutation was classified as clonal if its probability of being clonal was >50% or if the upper bound of the 95% confidence interval of its CCF was 100% (Landau et al., 2013).

Mutational signature analysis and mappability

We performed mutational signature analysis using the R package deconstructSigs (Rosenthal et al., 2016) as previously described (Ng et al., 2017b). First, the fraction of mutations found in each of the 96 possible trinucleotide contexts was calculated to build the mutational profile for each sample, normalized by the number of times each trinucleotide context is observed in the sequencing regions. For the pooled analysis, mutations from all samples were combined to result in a "pooled sample" profile. Next, the mutation profile was reconstructed with minimum error by

iteratively inferring the weighted contribution of each of the 30 reference signatures. Definition and interpretation of mutational signatures were obtained from COSMIC (http://cancer.sanger.ac.uk/cosmic/signatures).

We assessed mappability of SNVs in the GRCh37/hg19 reference genome using the 75bp CRG Alignability track available in the UCSC genome browser (http://genome.ucsc.edu/cgi-bin/hgTrackUi?db=hg19&g=wgEncodeMapability). The CRG Alignability track displays how uniquely 75-mer sequences align to different regions of the genome (Derrien et al., 2012). The mappability score is defined as the reciprocal of the number of matches found in the genome. A mappability score of 1 is considered high as it indicates a unique match.

To assess the effects of sequencing alignment using the newer BWA mem algorithm (http://bio-bwa.sourceforge.net/bwa.shtml) and of read length on variant identification in regions of low mappability, we obtained 10 tumor-normal pairs from The Cancer Genome Atlas breast cancer cohort (Kobolt et al., 2012a). These 10 tumor-normal pairs were sequenced using the 100bp paired-end sequencing. We 1) trimmed the raw sequences to 75bp and aligned the data with BWA aln (Li and Durbin, 2009), as we performed for our 6 cases in our manuscript, 2) trimmed the raw sequences to 75bp and aligned the data with BWA mem (http://bio-bwa.sourceforge.net/bwa.shtml), and 3) aligned the 100bp reads using BWA mem, then downsampled the resulting BAM files to 75% such that the overall sequencing depth largely matched the first 2 processing approaches. Mutation calling was performed using the matched-normal pipeline. Mappability was computed as described above.

Clonal inference

We performed clonal inference using PyClone (Roth et al., 2014), using the read counts from high-depth Ampliseq sequencing for all validated homogeneous and heterogeneous mutations. Major and minor copy numbers for each mutation, as well as tumor purity, were defined using FACETS (as described above). Case 2 biorep C, for which purity could not be estimated by FACETS, was set to 0.2. 10,000 iterations of Markov Chain Monte Carlo sampling were performed with the first 1,000 iterations discarded as "burn-in". Clusters composed of single mutation were discarded.

To assess the impact of sequencing depth on clonal inference, we performed a downsampling analysis. Specifically, we downsampled the number of reads for each mutation, at increments of 2.5% up to 25%, then at 5% up to 95%, of the original depth. Each downsampling experiment was performed 20 times. The downsampled number of reads was used as input to PyClone and clonal inference performed as described above. Clonal inference error was computed in relation to the clusters inferred from no downsampling. Clonal inference error was calculated as 1-(the number of mutations in the same clusters as no downsampling/the total number of mutations). All somatic mutations were included, regardless if they would have been considered somatic at the reduced depth.

SUPPLEMENTAL REFERENCES

Carter, H., Chen, S., Isik, L., Tyekucheva, S., Velculescu, V.E., Kinzler, K.W., Vogelstein, B., and Karchin, R. (2009). Cancer-specific high-throughput annotation of somatic mutations: computational prediction of driver missense mutations. Cancer Res. *69*, 6660–6667.

Carter, S.L., Cibulskis, K., Helman, E., McKenna, A., Shen, H., Zack, T., Laird, P.W., Onofrio, R.C., Winckler, W., Weir, B.A., et al. (2012). Absolute quantification of somatic DNA alterations in human cancer. Nat. Biotechnol. *30*, 413–421.

Chang, M.T., Asthana, S., Gao, S.P., Lee, B.H., Chapman, J.S., Kandoth, C., Gao, J., Socci, N.D., Solit, D.B., Olshen, A.B., et al. (2016). Identifying recurrent mutations in cancer reveals widespread lineage diversity and mutational specificity. Nat. Biotechnol. *34*, 155–163.

Choi, Y., Sims, G.E., Murphy, S., Miller, J.R., and Chan, A.P. (2012). Predicting the functional effect of amino acid substitutions and indels. PLoS One *7*, e46688.

Cibulskis, K., Lawrence, M.S., Carter, S.L., Sivachenko, A., Jaffe, D., Sougnez, C., Gabriel, S., Meyerson, M., Lander, E.S., and Getz, G. (2013). Sensitive detection of somatic point mutations in impure and heterogeneous cancer samples. Nat. Biotechnol. *31*, 213–219.

Cingolani, P., Platts, A., Wang, L.L., Coon, M., Nguyen, T., Wang, L., Land, S.J., Lu, X., and Ruden, D.M. (2012) A program for annotating and predicting the effects of single nucleotide polymorphisms, SnpEff. Fly, *6*, 80-92.

Dang, V.T., Kassahn, K.S., Marcos, A.E., and Ragan, M.A. (2008). Identification of human haploinsufficient genes and their genomic proximity to segmental duplications. Eur. J. Hum. Genet. *16*, 1350–1357.

De Mattos-Arruda, L., Weigelt, B., Cortes, J., Won, H.H., Ng, C.K.Y., Nuciforo, P., Bidard, F.-C., Aura, C., Saura, C., Peg, V., et al. (2014). Capturing intra-tumor genetic heterogeneity by de novo mutation profiling of circulating cell-free tumor DNA: a proof-of-principle. Ann. Oncol. *25*, 1729–1735.

Derrien, T., Estellé, J., Sola, S.M., Knowles, D.G., Raineri, E., Guigó, R., and Ribeca, P. (2012). Fast Computation and Applications of Genome Mappability. PLoS One *7*, e30377.

Futreal, P.A., Andrew Futreal, P., Lachlan, C., Mhairi, M., Thomas, D., Timothy, H., Richard, W., Nazneen, R., and Stratton, M.R. (2004a). A census of human cancer genes. Nat. Rev. Cancer *4*, 177–183.

Futreal, P.A., Andrew Futreal, P., Lachlan, C., Mhairi, M., Thomas, D., Timothy, H., Richard, W., Nazneen, R., and Stratton, M.R. (2004b). A census of human cancer genes. Nat. Rev. Cancer *4*, 177–183.

Kandoth, C., Cyriac, K., McLellan, M.D., Fabio, V., Kai, Y., Beifang, N., Charles, L., Mingchao, X., Qunyuan, Z., McMichael, J.F., et al. (2013a). Mutational landscape and significance across 12 major cancer types. Nature *502*, 333–339.

Kandoth, C., Cyriac, K., McLellan, M.D., Fabio, V., Kai, Y., Beifang, N., Charles, L., Mingchao, X., Qunyuan, Z., McMichael, J.F., et al. (2013b). Mutational landscape and significance across 12 major cancer types. Nature *502*, 333–339.

Koboldt, D.C., Fulton, R.S., McLellan, M.D., Schmidt, H., Kalicki-Veizer, J., McMichael, J.F., Fulton, L.L., Dooling, D.J., Ding, L., Mardis, E.R., et al. (2012a). Comprehensive molecular portraits of human breast tumours. Nature *490*, 61–70.

Koboldt, D.C., Zhang, Q., Larson, D.E., Shen, D., McLellan, M.D., Lin, L., Miller, C.A., Mardis, E.R., Ding, L., and Wilson, R.K. (2012b). VarScan 2: somatic mutation and copy number alteration discovery in cancer by exome sequencing. Genome Res. *22*, 568–576.

Landau, D.A., Carter, S.L., Stojanov, P., McKenna, A., Stevenson, K., Lawrence, M.S., Sougnez, C., Stewart, C., Sivachenko, A., Wang, L., et al. (2013). Evolution and impact of subclonal mutations in chronic lymphocytic leukemia. Cell *152*, 714–726.

Lawrence, M.S., Stojanov, P., Mermel, C.H., Robinson, J.T., Garraway, L.A., Golub, T.R., Meyerson, M., Gabriel, S.B., Lander, E.S., and Getz, G. (2014a). Discovery and saturation analysis of cancer genes across 21 tumour types. Nature *505*, 495–501.

Lawrence, M.S., Stojanov, P., Mermel, C.H., Robinson, J.T., Garraway, L.A., Golub, T.R., Meyerson, M., Gabriel, S.B., Lander, E.S., and Getz, G. (2014b). Discovery and saturation analysis of cancer genes across 21 tumour types. Nature *505*, 495–501.

Levandowsky, M., Michael, L., and David, W. (1971). Distance between Sets. Nature *234*, 34–35.

Li, H., and Durbin, R. (2009). Fast and accurate short read alignment with Burrows-Wheeler transform. Bioinformatics *25*, 1754–1760.

McKenna, A., Hanna, M., Banks, E., Sivachenko, A., Cibulskis, K., Kernytsky, A., Garimella, K., Altshuler, D., Gabriel, S., Daly, M., et al. (2010). The Genome Analysis Toolkit: a MapReduce framework for analyzing next-generation DNA sequencing data. Genome Res. *20*, 1297–1303.

Ng, C.K.Y., Bidard, F.-C., Piscuoglio, S., Geyer, F.C., Lim, R.S., de Bruijn, I., Shen, R., Pareja, F., Berman, S.H., Wang, L., et al. (2017a). Genetic Heterogeneity in Therapy-Naïve Synchronous Primary Breast Cancers and Their Metastases. Clin. Cancer Res. *23*, 4402–4415.

Ng, C.K.Y., Piscuoglio, S., Geyer, F.C., Burke, K.A., Pareja, F., Eberle, C., Lim, R., Natrajan, R., Riaz, N., Mariani, O., et al. (2017b). The Landscape of Somatic Genetic Alterations in Metaplastic Breast Carcinomas. Clin. Cancer Res. *23*, 3859–3870.

Qi, Y., Liu, X., Liu, C.-G., Wang, B., Hess, K.R., Symmans, W.F., Shi, W., and Pusztai, L. (2015). Reproducibility of Variant Calls in Replicate Next Generation Sequencing Experiments. PLoS One *10*, e0119230.

Robinson, J.T., Helga, T., Wendy, W., Mitchell, G., Lander, E.S., Gad, G., and Mesirov, J.P. (2011). Integrative genomics viewer. Nat. Biotechnol. *29*, 24–26.

Rosenthal, R., McGranahan, N., Herrero, J., Taylor, B.S., and Swanton, C. (2016). DeconstructSigs: delineating mutational processes in single tumors distinguishes DNA repair deficiencies and patterns of carcinoma evolution. Genome Biol. *17*, 31.

Roth, A., Khattra, J., Yap, D., Wan, A., Laks, E., Biele, J., Ha, G., Aparicio, S., Bouchard-Cote, A., Shah, S.P. (2014). PyClone: statistical inference of clonal population structure in cancer. Nat. Methods *11*, 396-398.

Saunders, C.T., Wong, W.S.W., Swamy, S., Becq, J., Murray, L.J., and Cheetham, R.K. (2012). Strelka: accurate somatic small-variant calling from sequenced tumor-normal sample pairs. Bioinformatics *28*, 1811–1817.

Schwarz, J.M., Christian, R., Markus, S., and Dominik, S. (2010). MutationTaster evaluates disease-causing potential of sequence alterations. Nat. Methods *7*, 575–576.

Shen, R., and Seshan, V.E. (2016). FACETS: allele-specific copy number and clonal heterogeneity analysis tool for high-throughput DNA sequencing. Nucleic Acids Res. *44*, e131.

Shihab, H.A., Gough, J., Cooper, D.N., Stenson, P.D., Barker, G.L.A., Edwards, K.J., Day, I.N.M., and Gaunt, T.R. (2013). Predicting the functional, molecular, and phenotypic consequences of amino acid substitutions using hidden Markov models. Hum. Mutat. *34*, 57–65.

Yang, H., and Wang, K. (2015). Genomic variant annotation and prioritization with ANNOVAR and wANNOVAR. Nat. Protoc. *10*, 1556–1566.

Zook, J.M., Brad, C., Jason, W., David, M., Oliver, H., Winston, H., and Marc, S. (2014). Integrating human sequence data sets provides a resource of benchmark SNP and indel genotype calls. Nat. Biotechnol. *32*, 246–251.
