# Supplementary material for: Reliability of Whole-Exome Sequencing for Assessing Intratumor Genetic Heterogeneity
Source: Cell Rep. Author manuscript; Available in PMC 2018 Nov 28. (PMC6261536; doi:10.1016/j.celrep.2018.10.046)
Supplement: 6 [file NIHMS1512150-supplement-6.pdf]

Cell Reports

Article

Reliability of Whole-Exome Sequencing for Assessing Intratumor Genetic Heterogeneity

Graphical Abstract

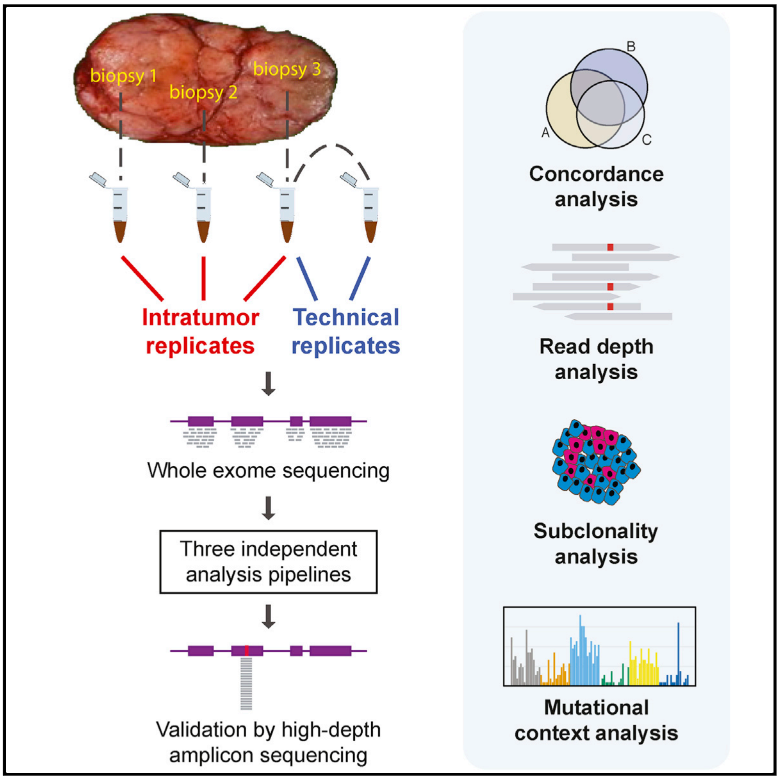

Authors

Weiwei Shi, Charlotte K.Y. Ng, Raymond S. Lim, ..., Lajos Pusztai, Jorge S. Reis-Filho, Christos Hatzis

Correspondence

reisfilj@mskcc.org (J.S.R.-F.), christos.hatzis@yale.edu (C.H.)

In Brief

Shi et al. report that standard coverage whole-exome sequencing and bioinformatics pipelines cannot discriminate between genuine intratumor genetic heterogeneity and sequencing artifacts. Although aggressive minimum depth filtering would not improve the false detection rate of subclonal mutations, excluding mutations in low-mappability regions or in certain mutational contexts could help.

Highlights

- Genuine intratumor genetic heterogeneity is hard to distinguish from sequencing artifacts
- Cancer-only WES pipelines are unreliable (69% somatic mutations are false positive)
- 34%–80% of somatic variants contributing to genetic heterogeneity are technical noise
- Excluding mutations in low-mappability regions and higher coverage are needed

Shi et al., 2018, Cell Reports *25*, 1446–1457
November 6, 2018 © 2018 The Author(s).
https://doi.org/10.1016/j.celrep.2018.10.046

OPEN ACCESS
CellPress

Cell Reports
Article

Reliability of Whole-Exome Sequencing for Assessing Intratumor Genetic Heterogeneity

Weiwei Shi,[1,11] Charlotte K.Y. Ng,[2,3,4,11] Raymond S. Lim,[2,11] Tingting Jiang,[1] Sushant Kumar,[5,6] Xiaotong Li,[1,6] Vikram B. Wali,[1] Salvatore Piscuoglio,[2,3] Mark B. Gerstein,[5,6,7] Anees B. Chagpar,[8,9] Britta Weigelt,[2] Lajos Pusztai,[1,9] Jorge S. Reis-Filho,[2,10,*] and Christos Hatzis[1,9,12,*]

[1]Department of Medicine, Yale School of Medicine, Yale University, New Haven, CT, USA
[2]Department of Pathology, Memorial Sloan Kettering Cancer Center, New York, NY, USA
[3]Institute of Pathology, University Hospital Basel, Basel, Switzerland
[4]Department of Biomedicine, University of Basel, Basel, Switzerland
[5]Molecular Biophysics and Biochemistry, Yale University, New Haven, CT, USA
[6]Program in Computational Biology and Bioinformatics, Yale University, New Haven, CT, USA
[7]Computer Science, Yale University, New Haven, CT, USA
[8]Department of Surgery, Yale School of Medicine, Yale University, New Haven, CT, USA
[9]Yale Cancer Center, New Haven, CT, USA
[10]Human Oncology and Pathogenesis Program, Memorial Sloan Kettering Cancer Center, New York, NY, USA
[11]These authors contributed equally
[12]Lead Contact
*Correspondence: reisfilj@mskcc.org (J.S.R.-F.), christos.hatzis@yale.edu (C.H.)
https://doi.org/10.1016/j.celrep.2018.10.046

SUMMARY

Multi-region sequencing is used to detect intratumor genetic heterogeneity (ITGH) in tumors. To assess whether genuine ITGH can be distinguished from sequencing artifacts, we performed whole-exome sequencing (WES) on three anatomically distinct regions of the same tumor with technical replicates to estimate technical noise. Somatic variants were detected with three different WES pipelines and subsequently validated by high-depth amplicon sequencing. The cancer-only pipeline was unreliable, with about 69% of the identified somatic variants being false positive. Even with matched normal DNA for which 82% of the somatic variants were detected reliably, only 36%–78% were found consistently in technical replicate pairs. Overall, 34%–80% of the discordant somatic variants, which could be interpreted as ITGH, were found to constitute technical noise. Excluding mutations affecting low-mappability regions or occurring in certain mutational contexts was found to reduce artifacts, yet detection of subclonal mutations by WES in the absence of orthogonal validation remains unreliable.

INTRODUCTION

Intratumor genetic heterogeneity (ITGH), typically defined as the coexistence of genetically distinct but clonally related cancer cells within the same patient (Yap et al., 2012), can manifest itself spatially within the same lesion or as genetic differences between different metastatic sites and the primary tumor from the same patient (Ding et al., 2010; Gerlinger et al., 2012; Marusyk et al., 2012; Newburger et al., 2013; Yates et al., 2015). The broad availability of massively parallel sequencing has accelerated research into ITGH, and numerous studies have applied whole-exome or targeted-exome sequencing to multiple biopsies from the same cancer, to different metastatic lesions from the same patient, and more recently to multiple single cells from the same cancer (Gerlinger et al., 2012; Hou et al., 2012; Martelotto et al., 2017; Navin et al., 2011; Newburger et al., 2013; Nik-Zainal et al., 2012; Wang et al., 2014; Xu et al., 2012). ITGH represents a snapshot of the tumor's evolutionary path and is a clinically important phenomenon with implications in prognosis and treatment response (Fisher et al., 2013; Hiley et al., 2014; Jiang et al., 2014; Marusyk et al., 2012; Morris et al., 2016; Turner and Reis-Filho, 2012).

The assessment of ITGH, by definition, involves the detection of subclonal, low-frequency variants that are not uniformly present in all cancer cells and is made possible by the availability of bioinformatics tools to detect low-frequency somatic mutations with high sensitivity (Cibulskis et al., 2013; Koboldt et al., 2012; Saunders et al., 2012; Wilm et al., 2012). However, the presence of technical noise in sequencing data is well known (Li, 2014; Nakamura et al., 2011), and it is unclear whether genuine ITGH can be reliably distinguished from artifacts generated during library preparation, sequencing, and data processing (Qi et al., 2015; Smith et al., 2014). Understanding the signal-to-noise characteristics in these experiments is critical for the interpretation of ITGH.

Given the implications in prognosis and treatment response, ITGH is an important consideration in the clinical setting. Because the cost of WES and complex informed consent requirements, the inclusion of matching normal samples still represents a limitation in the sequencing of tumor samples in some large clinical trials (Shi et al., 2017). Whether subclonal mutations can be robustly identified in the absence of matching normal samples and whether pooled normal samples from unrelated

This is an open access article under the CC BY license (http://creativecommons.org/licenses/by/4.0/).

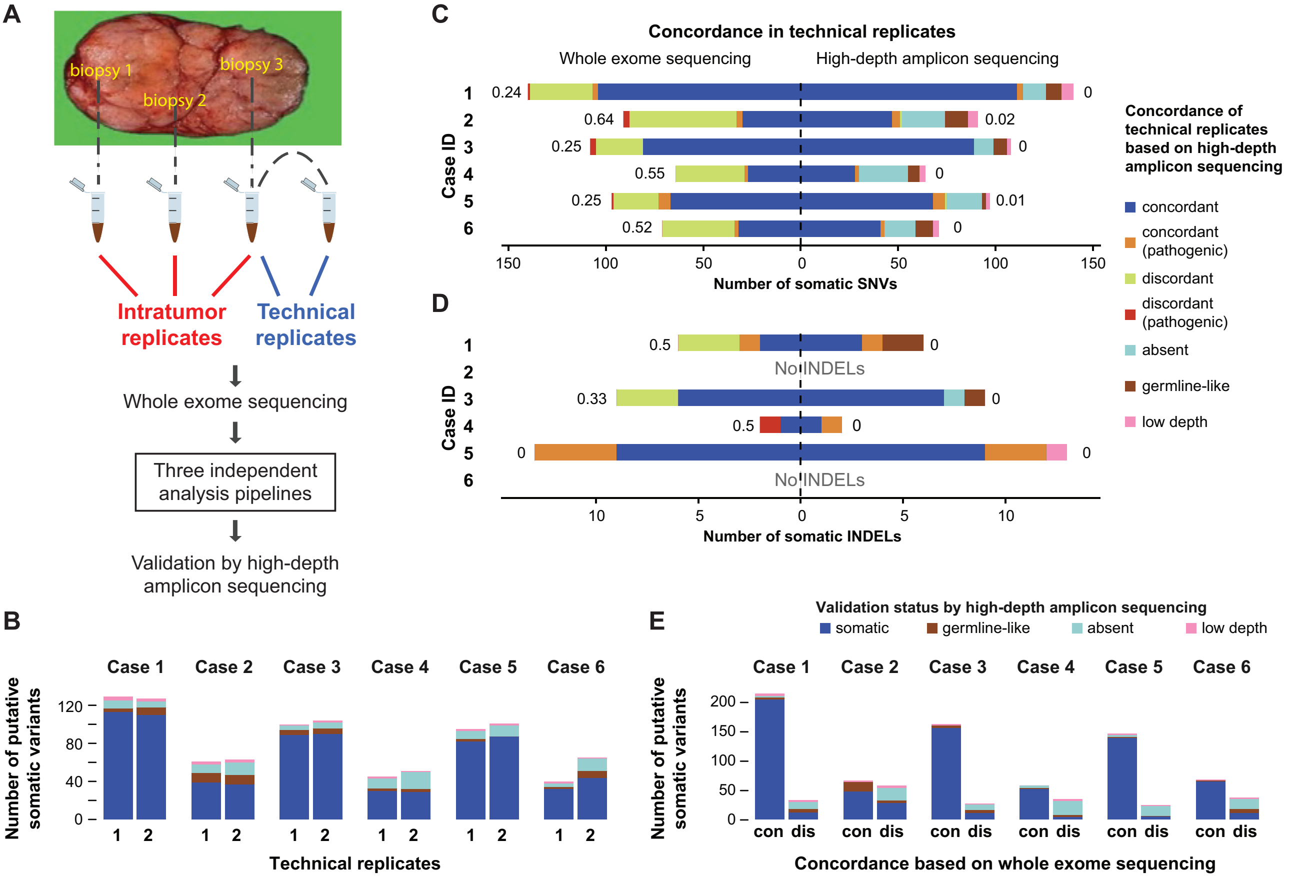

Figure 1. Reliability of Somatic SNVs and INDELs Detected by WES and High-Depth Amplicon Sequencing Validation of Variants

(A) Biopsies were obtained from three anatomically distinct regions of each tumor to assess spatial genomic heterogeneity. One of the three DNA samples was split in two to provide a pair of technical replicates. Somatic variants were detected by three different WES analysis pipelines and subsequently validated by high-depth amplicon sequencing.

(B) Number of somatic mutations identified by WES in each technical replicate, subclassified according to their validation status by high-depth amplicon sequencing.

(C and D) Concordance of somatic SNVs (C) and INDELs (D) defined in each pair of technical replicates using the matched-normal WES analysis pipeline (left) or high-depth amplicon sequencing (right). Discordance between the replicates quantified as the Jaccard distance shown next to each bar and the pathogenicity of variants were assessed as described in Supplemental Experimental Procedures. Putative WES variants re-sequenced with high-depth amplicon sequencing were further classified as absent (VAF < 1%), germline (tumor VAF/germline VAF < 5), or low depth (<50×).

(E) Validation status of variants (SNVs and INDELs) detected by WES in technical replicate pairs, categorized as concordant (“con”) or discordant (“dis”) by WES, and the distribution of their AmpliSeq validation status is shown within each bar.

See also Figure S1.

individuals would serve as a reasonable control should be explored as alternative options for the assessment of ITGH in the clinical setting.

In this study, we aimed to assess the reliability of somatic variant detection from whole-exome sequencing (WES) in the context of ITGH (Figures 1A and S1A). To address this question, we performed WES on DNA from biopsies obtained from three anatomically distinct regions of six primary breast cancers (6 × 3 biopsies) and from matched peripheral blood leukocytes. Additionally, to determine the background noise levels as a comparator for the assessment of ITGH, aliquots of the same DNA samples from six distinct biopsies were sequenced twice to generate technical replicates. We examined the reliability of somatic variant detection using three different analysis approaches, namely, using cancer WES data only, cancer data and WES from pooled unrelated (i.e., non-matched) normal samples, and WES data from cancer and matching normal tissue (i.e., blood). Each approach used different sets of detection algorithms and filtering steps in an attempt to control for the specific biases associated with each approach. To generate the “gold standard” benchmark dataset, we re-sequenced all somatic variants identified by any of the three somatic variant detection pipelines in at least one sample using high-depth targeted amplicon sequencing. Given the higher depth obtained by amplicon sequencing, we assessed true ITGH and estimated the frequency of false positives by the different detection pipelines. Finally, we evaluated the sequence patterns and context in which the artifactual

Table 1. Tumor Characteristics and Estimated Tumor Cellularity and Ploidy from WES Data Using FACETS

Sample	Subtype	Stage	Grade	Estimated Purity (%)	Estimated Ploidy
Case 1 biorep A/techrep 1	ER+/PR+/ HER2+	IIA	2	53.8	1.92
Case 1 biorep B				46.3	2.00
Case 1 biorep C				26.0	1.92
Case 1 techrep 2				54.0	1.92
Case 2 biorep A	ER−/PR−/ HER2−	IIB	3	40.0	2.14
Case 2 biorep B/techrep 1				32.3	1.79
Case 2 biorep C				NE	1.72
Case 2 techrep 2				38.5	2.03
Case 3 biorep A	ER+/PR+/ HER2+	IIB	2	56.4	1.89
Case 3 biorep B				72.8	1.93
Case 3 biorep C/techrep 1				64.9	1.90
Case 3 techrep 2				66.7	1.93
Case 4 biorep A	ER+/PR−/ HER2−	IIB	2	60.4	1.98
Case 4 biorep B/techrep 1				61.7	1.95
Case 4 biorep C				57.6	1.99
Case 4 techrep 2				57.6	1.94
Case 5 biorep A	ER−/PR−/ HER2−	IIA	3	78.7	2.60
Case 5 biorep B/techrep 1				76.7	2.62
Case 5 biorep C				80.8	2.82
Case 5 techrep 2				76.2	2.61
Case 6 biorep A/techrep 1	ER+/PR+/ HER2+	IIA	2	47.8	1.99
Case 6 biorep B				49.4	2.05
Case 6 biorep C				52.9	2.18
Case 6 techrep 2				49.0	1.96

biorep, biological replicate; NE, could not be estimated; techrep, technical replicate; WES, whole-exome sequencing.

mutations occurred to improve the specificity of WES for characterizing genuine ITGH.

RESULTS

Limited Reliability of Somatic Mutations Defined by WES

We performed WES on DNA extracted from three distinct regions of the primary tumor and the matching normal blood cells from six breast cancer patients, including four with estrogen receptor-positive and two with triple-negative cancers (Table 1). The biopsies were obtained from three anatomically distinct regions of each tumor at least 1 cm apart (i.e., intratumor replicates; Figure 1A) in the context of a prospective institutional review board-approved study to assess intratumor molecular heterogeneity (von Wahlde et al., 2017). All tumor samples had at least 50% tumor cellularity on the basis of pathologic assessment. For the technical replicates, a second library was generated from one of the tumor DNA samples randomly selected from each cancer and sequenced by WES using the same protocol at the same facility on a different day. The mean target depth was 160× (range 70× to 220×; Table S1), consistent with recommendations for WES (Clark et al., 2011; Sims et al., 2014). Following WES, somatic single-nucleotide variants (SNVs) and small insertion-deletions (INDELs) were identified by three different somatic variant calling pipelines that used (1) the tumor DNA alone (i.e., tumor only, a common approach in clinical practice), (2) tumor DNA and pooled unrelated normal DNA (i.e., cohort normal), or (3) tumor DNA and patient-matched normal DNA (i.e., matched normal; Figure S1A; Supplemental Experimental Procedures). Targeted amplicon sequencing using an orthogonal library generation and an independent sequencing method (AmpliSeq) was performed for all putative somatic variants identified by any of the WES variant calling pipelines on all tumor and matching normal DNA to a median depth of 605× to define the "gold standard" mutation status for each identified somatic variant (Table S1; Supplemental Experimental Procedures).

To quantify the technical reliability of somatic mutation detection by the matched-normal WES pipeline, the approach that is most frequently used in the research setting to assess ITGH, we compared the somatic mutations identified in the six pairs of technical replicates. In this experiment, tumor samples and normal samples were sequenced to a median coverage of 184× (range 92–211) and 90× (range 80–138), respectively (Table S1). We identified medians of 74 (range 40–125) and 3 (range 0–13) somatic SNVs and INDELs, respectively, in each of the 12 DNA samples. Considering the high-depth amplicon sequencing results as the "gold standard," we categorized candidate mutations detected in WES as true somatic, absent, germline-like (incorporating genuine germline variants and artifactual variant alleles caused by alignment biases and/or the sequencing technology) (Kim and Speed, 2013), or low-depth (i.e., technical failure with amplicon sequencing; Table S2; Supplemental Experimental Procedures). Excluding the low-depth (technical failure) variants, a median 82% (range 56%–90% per sample) of the somatic SNVs were confirmed as somatic, a median 5% (range 0%–16%) as germline-like, and the remaining were absent by AmpliSeq (Figure 1B; Table S3).

Given that both technical replicates used the same input DNA, we anticipated detecting nearly identical somatic mutations in each pair of replicates. However, only a subset of the somatic SNVs and INDELs were consistently identified in technical replicate pairs, with median Jaccard distances (ranging from 0, perfect agreement, to 1, absence of overlapping variants; Supplemental Experimental Procedures) of 0.39 (range 0.24–0.64) for SNVs (Figure 1C) and 0.17 (range 0–0.50) for INDELs (Figure 1D). Interestingly, the technical replicate pairs with the highest Jaccard distances were those with the lowest tumor cell content as inferred by FACETS (Shen and Seshan, 2016) (Table 1). There were also a small number of potentially pathogenic variants among the discordant variants in technical replicate pairs (range 0–3; Figure 1C, red bars) that could have

been misinterpreted as ITGH. We obtained fewer somatic mutations but similarly modest reproducibility with the cohort-normal WES pipeline and far fewer somatic mutations but improved reproducibility using the tumor-only pipeline (see explanation below; Figures S1B and S1C). Comparing only the mutations that were confirmed to be somatic by AmpliSeq between the technical replicate pairs, we observed almost perfect agreement for SNVs (Figure 1C) and for INDELs (Figure 1D) (maximum Jaccard distance of 0.02 and 0, respectively).

When we examined the somatic mutations found to be concordant or discordant between pairs of technical replicates on the basis of WES, a median of 95% (range 73%–97%) of the concordant variants were confirmed to be genuinely somatic in at least one of the two technical replicates, compared with a median 33% (range 14%–48%) of the discordant variants (Figure 1E). Of the discordant WES variants, a median of 44% (range 36%–71%) were found to be absent by AmpliSeq (i.e., false positive in one of the two technical replicates), and a median of 7% (range 3%–22%) were germline(-like) variants (i.e., missed by WES in the matching normal). The validation status of 3% (range 1%–8%) of the mutations could not be ascertained, because of technical failure of low AmpliSeq coverage in the validation experiments.

Taken together, these results suggest that WES performed at typical sequencing depth may be inadequate for detecting ITGH, particularly when the tumor cell content is less than 50%, as only 62% (range 36%–76%) of the somatic mutations were detected consistently in the technical replicate pairs by WES, with the remaining mutations falsely appearing as discordant.

WES Overestimates True ITGH

Next, we quantified ITGH by comparing somatic variant calls between the geographically distinct biopsies from the same cancer. In this analysis, we also examined how the three different WES analytic approaches differed in the quantification of ITGH (Figure S1A; Supplemental Experimental Procedures). Tumor cellularity of all samples was inferred from WES using FACETS (Shen and Seshan, 2016) (Table 1). We used mixed-effects linear modeling to estimate an average cellularity of 54.7% with intratumor and technical SDs of 7.0% and 2.2%, respectively (Supplemental Experimental Procedures). The matched-normal pipeline detected a median of 150 unique somatic mutations (range 68–186) in each tumor. Compared with the matched-normal pipeline, the cohort-normal pipeline detected a median of 101 mutations (range 48–131; $p > 0.05$, Wilcoxon test), and the tumor-only pipeline identified a median of 62 mutations (range 36–97; $p = 0.01$, Wilcoxon test; Figures 2A and 2B; Table S4).

To assess the reliability of the different WES pipelines in detecting ITGH, we compared the WES candidate mutations with the "gold-standard" AmpliSeq-validated somatic variants from the same sample. A median of 62% (range 50%–98%) of the candidate somatic variants detected by the tumor-only pipeline were germline variants, and only 28% (range 2%–45%) were validated as true somatic mutations, highlighting the challenges posed by this commonly used approach in clinical practice. By contrast, a median of 79% (range 59%–91%) and 84% (range 61%–92%) of the variants defined by the cohort-normal and the matched-normal pipelines, respectively, were true somatic mutations (Figures 2A, S1D, and S1E). The tumor-only pipeline had the lowest sensitivity (median 18%, range 0%–29%) and precision (median 28%, range 0%–46%) for identifying true somatic variants. The matched-normal pipeline had the highest sensitivity (median 87%, range 47%–94%) and precision (median 86%, range 62%–94%; Figure 2C), whereas the cohort-normal pipeline had similar precision (median 83%, range 61%–91%) but significantly lower sensitivity (median 48%, range 26%–64%; $p < 0.001$, Wilcoxon test). Using the matched-normal pipeline, tumor cellularity was positively correlated with sensitivity (Pearson $r = 0.7$, $p = 0.001$) and numerically, though not statistically significantly, correlated with precision (Figure S2A). We did not observe the same correlation with the other two pipelines.

Next, we estimated the apparent ITGH on the basis of mutations detected by the three WES calling pipelines using the Jaccard distance as the metric of ITGH. Mutations identified as somatic in one or two of the three biopsies and as germline-like or absent in the remaining biopsies contributed to ITGH. The tumor-only pipeline had a median Jaccard distance of 0.34 (range 0.19–0.96) compared with the cohort-normal pipeline of 0.70 (range 0.53–0.91) and matched-normal pipeline of 0.60 (range 0.44–0.89) (Figure 2B). The apparently lower ITGH defined by the tumor-only pipeline ($p = 0.03$ for tumor-only versus cohort-normal, $p > 0.05$ versus matched-normal, paired Wilcoxon tests) was due to the large number of germline variants misidentified as somatic mutations by the tumor-only pipeline (Figure 2A). When ITGH was estimated on the basis of the AmpliSeq-validated somatic mutations only, the median Jaccard distance was 0.40 (range 0.19–0.61; Figure 2B), which in the context of this study was considered a true estimation of ITGH. We also observed that the proportion of private mutations in a given biopsy was positively correlated with its purity relative to the mean purity of all biopsies for the patient (Pearson $r = 0.531$, $p = 0.023$; Figure S2B), suggesting that ITGH may be overestimated in cases with large variability in tumor purity between biopsies. Compared with the apparent ITGH defined by the candidate mutations in the cohort-normal and matched-normal WES pipelines, the true ITGH was significantly smaller ($p = 0.015$ versus cohort-normal and $p = 0.015$ versus matched-normal, paired Wilcoxon tests). We noted that 5.8% of the heterogeneous variants, also called branch mutations, that were not present in all biopsies of a given case were predicted to be pathogenic, but there was no statistically significant enrichment in pathogenic mutations compared to non-pathogenic variants among the heterogeneous somatic variants (Figure 2B). Heterogeneous variants were detected in a small number of cancer genes (21 of 306 [6.9%]), and five of these variants (1.6%) were predicted pathogenic.

Taken together, these results suggest that the tumor-only WES pipeline misidentifies a substantial proportion of germline variants as somatic mutations. Even when using the matched-normal DNA for mutation detection, the extent of ITGH defined solely on the basis of WES performed at typical sequencing depth is overestimated, potentially affecting actionable cancer

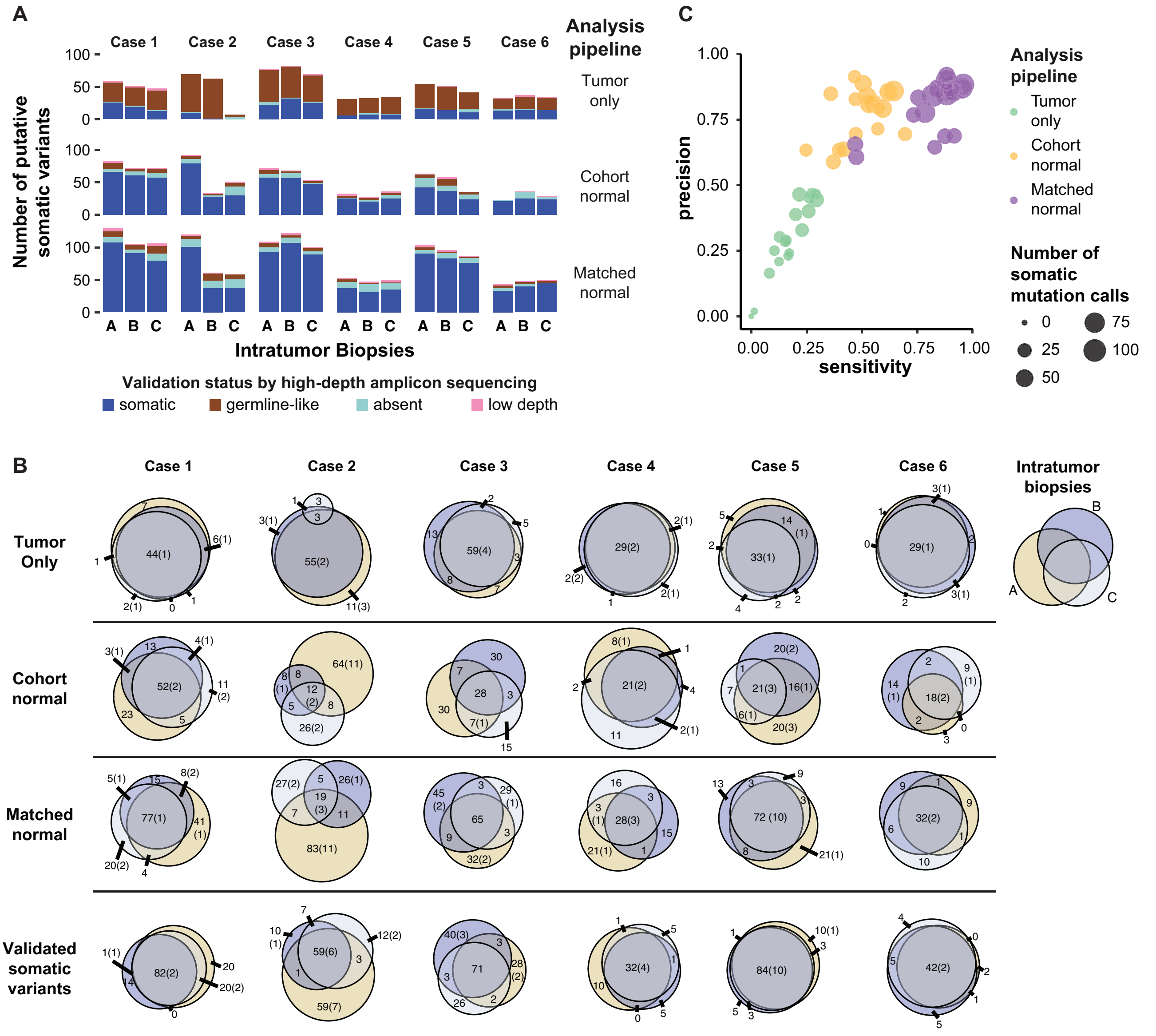

Figure 2. ITGH as Assessed by Different WES Analysis Pipelines

(A) Total number of somatic variants (SNVs and INDELs) identified in intratumor biopsies by the three WES analysis pipelines. One of the two technical replicates was randomly selected for inclusion in this analysis. Validation status by high-depth amplicon sequencing (i.e., somatic, germline, absent [VAF < 1%], low depth [< 50× coverage]) is shown according to the color key.

(B) Venn diagrams showing the overlap of putative somatic variants detected in intratumor biopsies from each tumor by the three WES analysis pipelines. The last row includes only "true" somatic variants validated by high-depth amplicon sequencing. The size of the circles is proportional to the number of somatic variants in a biopsy, with the numbers representing the total variants and those in parentheses indicating the number of pathogenic variants.

(C) Performance characteristics of the three WES analysis pipelines to identify true somatic variants. Putative somatic variants were considered as "true" if confirmed by high-depth amplicon sequencing. Precision was calculated as TP/(FP + TP) and sensitivity as TP/STP, where TP and FP are the number of true-positive and false-positive variants and STP is the total number of true somatic calls made by all three pipelines. Each circle represents one sample as analyzed by each pipeline, and the size of the circles is proportional to the number of putative somatic variants per biopsy identified by each analysis pipeline.

See also Figure S2.

genes. For example, a deleterious stop-gain branch mutation in *CDC27* (p.Cys71*) was identified as heterogeneous (in one of the three biopsies) by the matched-normal WES pipeline but was not validated by AmpliSeq. False-positive heterogeneous variants were mostly not actionable, however.

Characteristics of Artifactual WES Somatic Mutations

To identify the characteristics of the putative mutations identified by WES that were subsequently found not to be truly somatic variants, we examined the alternative coverage (i.e., the number of reads supporting the alternate allele), the variant allele

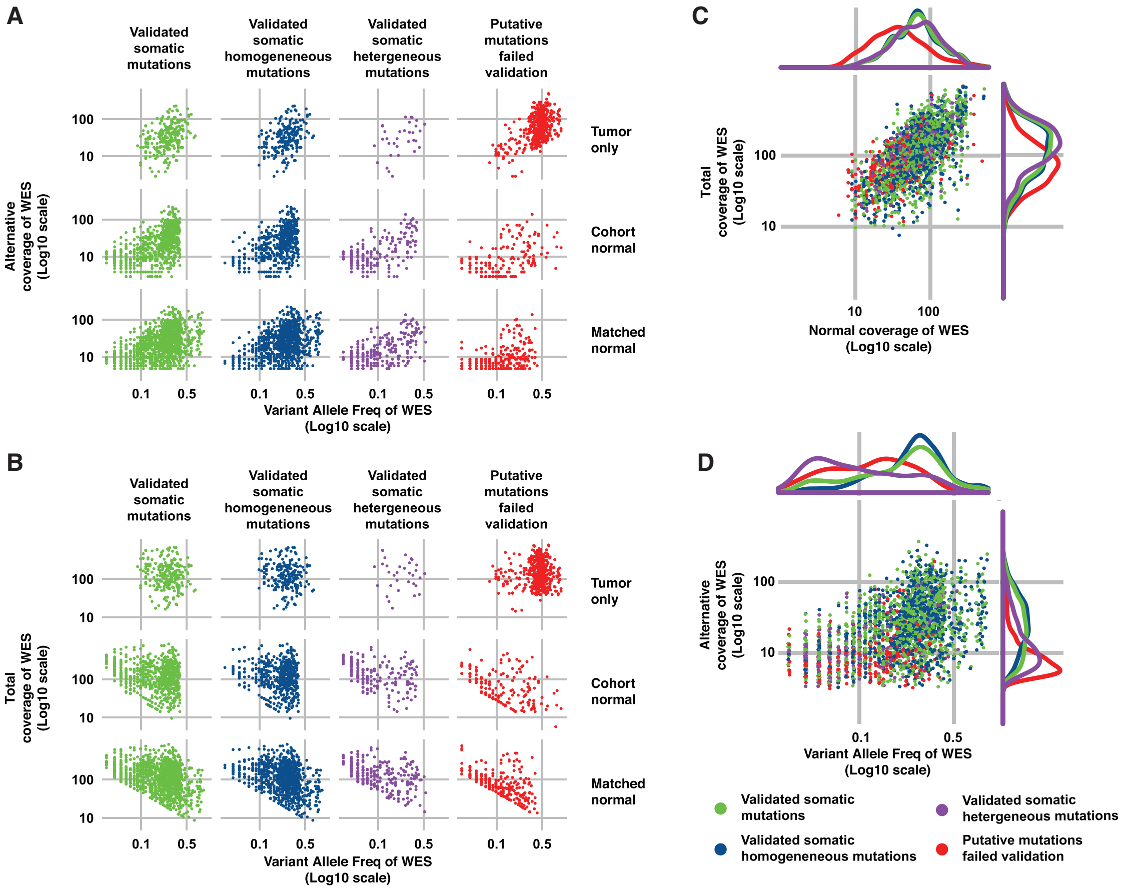

Figure 3. Coverage Characteristics of True Somatic Variants and False-Positive Mutations in the WES Data

(A and B) Alternative allele coverage (i.e., number of read supporting the alternative allele) (A) and total coverage (B) are plotted against variant allele fraction (VAF; all $\log_{10}$ scale) for the three WES analysis pipelines (rows). The different subsets of WES putative somatic variants according to validation status by high-depth amplicon sequencing are shown as columns: validated somatic mutations, validated homogeneous somatic mutations (i.e., present in all three biopsies of the same tumor), validated heterogeneous somatic mutations (i.e., present in one or two biopsies from the same tumor), and putative somatic mutations identified by the WES pipelines but failed validation by high-depth amplicon sequencing (i.e., putative somatic mutations that were validated to be germline, absent [VAF < 1%] or low coverage [< 50x]).

(C and D) For the matched-normal WES pipeline, (C) total coverage in the tumor (bottom) is plotted against the coverage in the matched normal sample, and (D) WES alternative allele coverage is plotted against VAF and of somatic mutations identified in all the specimens. The validation status categories are the same as in (A) and (B). Density kernel plots of the marginal distributions are included above and to the right of the scatterplots for each of the four categories of mutations. See also Figure S3.

frequency (VAF), and the total depth of coverage of the candidate somatic mutations identified by the three WES pipelines from the intratumor biopsies. The tumor-only pipeline reported a median of 2 variants (range 0–5) with VAF < 10% because of reduced sensitivity of the single-sample mutation detection algorithm at low VAF and the strict filtering imposed to remove potential germline variants (Figures 3A and 3B). Despite aggressive filtering, most of the putative somatic variants from the tumor-only analysis were germline variants with VAF ~ 50%, indicating the presence of a large number of private mutations that have not been cataloged in publicly available databases (Figures 2A, 3A, and 3B). The cohort-normal analysis correctly identified somatic variants with low VAF, including many that were heterogeneous between the biopsies, but missed somatic variants with VAF > 45% because of filtering imposed to remove likely germline variants that may not be present in the pooled normal DNA used as the reference (Figures 2C, 3A, and 3B). The validated somatic mutations defined by the matched-normal pipeline covered the widest range of VAFs. The putative somatic mutations identified by the matched-normal pipeline that were found to be absent by AmpliSeq were mostly in the low-VAF range (median 7.3%, range 0.6%–44%; Figures 2A, 3A, and 3B).

Given that the majority of the ITGH studies carried out to date (Ding et al., 2010; Gerlinger et al., 2012; Nik-Zainal et al., 2012) used matched tumor-normal samples and analysis pipelines similar to our matched-normal pipeline and that mainly low-VAF mutations contributed to ITGH (Figures 3A and 3B), we compared the true- and false-positive somatic mutations (i.e., the validated and the unvalidated putative somatic variants) and the validated homogeneous (i.e., present in all biopsies

from a case) and heterogeneous (i.e., absent in at least one biopsy from a case) somatic mutations derived from the distinct biopsies using the matched-normal WES pipeline. We found that the total depth for the true positive (median 124, range 9–1,028; Figure 3C, green) was significantly higher than for the false-positive mutations (median 78, range 12–926; $p < 0.001$, Wilcoxon test; Figures 3C and S3A, red). Furthermore, the false positives, compared with true mutations, had significantly lower alternative coverage (median 7, range 1–126 versus median 22, range 4–231; $p < 0.001$, Wilcoxon test) and VAF (median 11%, range 0.6%–64% versus median 23%, range 3%–93%; $p < 0.001$, Wilcoxon test; Figures 3D and S3B). There were also significant differences in the VAF distribution of the validated homogeneous and heterogeneous mutations (median 25%, range 1%–93% versus median 6%, range 1%–57%; $p < 0.001$, Wilcoxon test) and between the validated homogeneous mutations and the false positives (median 25%, range 1%–93% versus median 12%, range 2%–64%; $p < 0.001$, Wilcoxon test; Figures 3D and S3B). Crucially, the validated mutations implicated in true ITGH had significantly lower VAF than the false positives ($p < 0.001$, Wilcoxon test; Figures 3D and S3B–S3E), which indicates that true ITGH mutations may display similarly low or lower VAFs compared with the false-positive and false-negative mutations. These results suggest that filtering somatic variants with low VAF or low alternative coverage may improve the precision of the WES pipeline but would also eliminate many true somatic variants that contribute to ITGH. Importantly, false-positive mutations had significantly lower total depth in the matched normal DNA (median 35, range 6–492) compared with true-positive mutations (median 68, range 9–514; $p < 0.001$, Wilcoxon test) and validated heterogeneous mutations (median 72, range 12–343; $p < 0.001$, Wilcoxon test; Figure 3C). Many putative somatic mutations (47%) with total depth in the normal DNA of 10 or less were confirmed as germline-like by AmpliSeq, emphasizing the importance of having adequate sequencing depth in the normal samples.

Separating True ITGH from WES Artifacts

Because subclonal mutations are expected to be the predominant contributors to ITGH, we inferred the clonality of all mutations identified by WES using ABSOLUTE (Carter et al., 2012). Subclonal mutations were significantly overrepresented among the validated heterogeneous variants compared with the homogeneous somatic variants (83.2% versus 28.9%; $p < 0.001$, Fisher's exact test) but were similarly overrepresented among the artifactual somatic variants (91.5% versus 83.2%; $p > 0.05$, Fisher's exact test; Figures 4A and S4A). Importantly, subclonal mutations were also significantly enriched among discordant variants in the WES technical replicates compared with the concordant variants (72.0% versus 24.9%; $p < 0.001$, Fisher's exact test; Figures 4A and S4A). These results suggest that compared with clonal variants, subclonal variants detected by WES are more likely to be erroneously attributed to ITGH.

Examination of the genomic locations of mutations revealed that 41.1% of the artifactual somatic mutations occurred in regions of low mappability (Derrien et al., 2012) compared with only 6.4% for the validated somatic heterogeneous mutations ($p < 0.001$, Fisher's exact test; Figures 4B and S4B). Furthermore, 30.8% of the discordant variants in the WES technical replicates occurred in regions of low mappability compared with 8.4% for the concordant variants ($p < 0.001$, Fisher's exact test; Figures 4B and S4B). These results suggest that ambiguous mapping of DNA fragments directly contributes to artifactual somatic variants, even if longer reads (100 bp) were used (Figure S4C). Compared with the validated heterogeneous mutations, artifactual somatic mutations appeared to be significantly enriched in T > C transitions ($p < 0.001$, Fisher's exact test; Figures 4C and S5A), particularly in the ApTpA and NpTpG trinucleotide contexts and also in T > G transversions in the GpTpG context (Figure 4E). By contrast, artifactual somatic mutations were significantly depleted in C > G transversions ($p < 0.001$, Fisher's exact test; Figures 4C and S5A). Interestingly, validated somatic heterogeneous mutations were enriched for C > G substitutions in the TpCpA and TpCpT contexts (Figure 4E), the characteristic substitution patterns induced by the upregulation of APOBEC cytidine deaminases (Nik-Zainal et al., 2016). However, this enrichment appears to be driven primarily by the case with the largest variability in tumor purity (Figures S2B and S5A). Although a substantial proportion of the artifactual mutations detected were C > T transitions, which have been associated with both the aging process and with lab-induced cytosine deamination during DNA library preparation (Alexandrov et al., 2013; Chen et al., 2014), their proportion was similar between the artifactual and validated somatic heterogeneous variants (34.6% versus 33.9%; $p > 0.05$, Fisher's exact test; Figure 4C), with mutations occurring in the TpCpN context being significantly underrepresented in the artifactual variants (Figure 4E). We also did not observe an enrichment of C > T substitutions at low VAFs among the artifactual somatic mutations, which would have been indicative of likely FFPE fixation artifacts (Graw et al., 2015; Do and Dobrovic, 2015) (Figure S5B). Mutational signature analysis using previously defined mutational signatures (Alexandrov et al., 2013) identified signatures 5 and 29 to be overrepresented in the artifactual somatic mutations ($p = 0.028$, Wilcoxon test; Figure 4D), with a median of 16.1% (range 0%–44.4%) of the artifactual mutations classified as signature 5, driven mainly by the T > C transitions reported above. Signature 29 is driven by C > A mutations, predominantly in the ApCpA and GpCpA contexts, that were significantly overrepresented among the artifactual variants (Figure 4E).

Finally, we considered whether applying filtering strategies to exclude mutations that occur in low-mappability regions or within sequence contexts enriched in artifactual mutations (C > A mutations in ApCpA and GpCpA contexts, T > C mutations in ApTpA and [C/T/G]pTpG contexts, and T > G mutations in GpTpG context; Figure 4E) can improve the reliability of WES. Excluding putative somatic mutations in low-mappability regions improved the precision for mutations detected in the technical (0.862 versus 0.792 without filtering) and biological replicates (0.876 versus 0.817 without filtering), while reducing moderately the sensitivity (0.858 versus 0.913 for technical, 0.773 versus 0.824 for biological). Filtering by mutational context reduced the sensitivity without appreciably improving precision, as did filtering by the combination of filters (Figure 5).

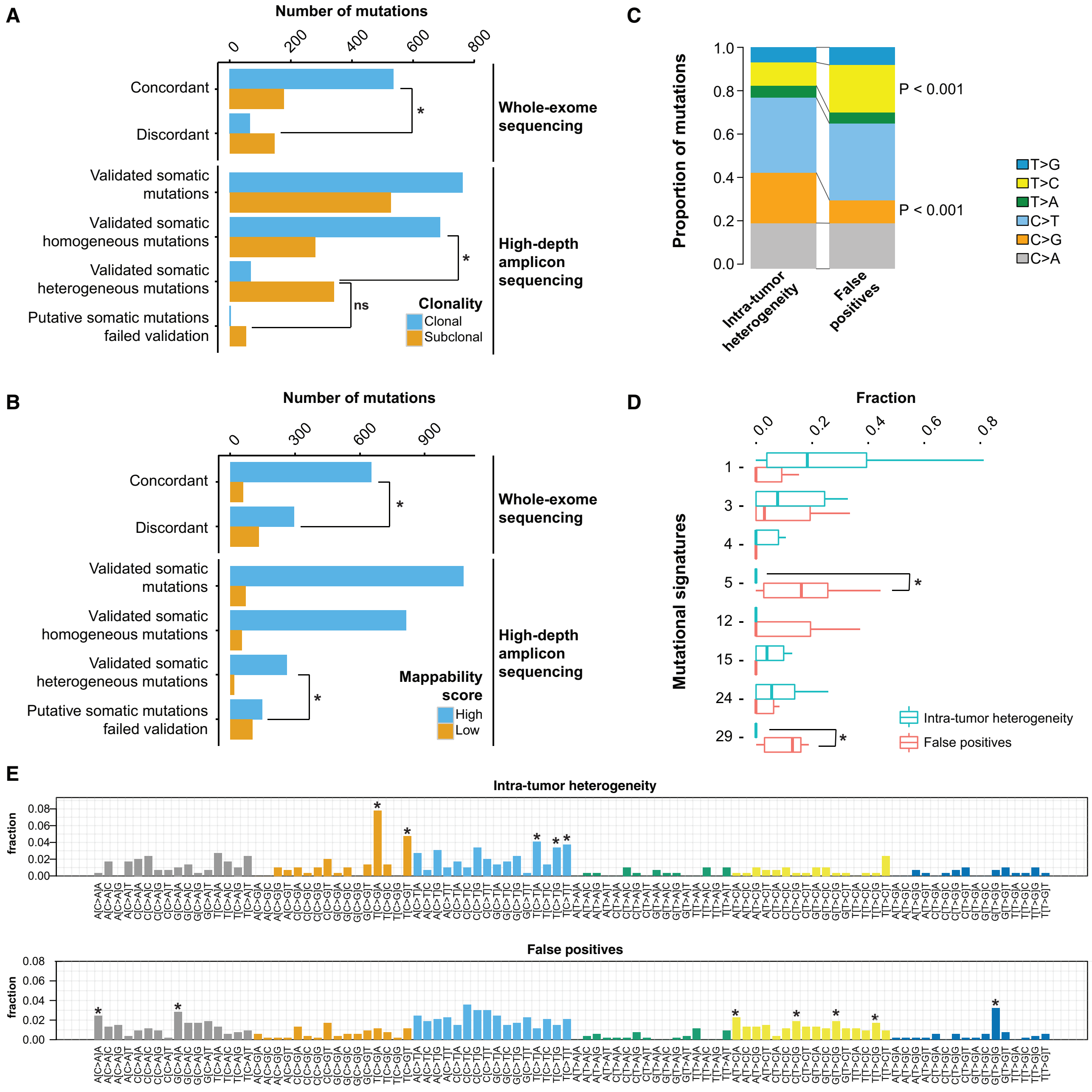

Figure 4. Mappability and Sequence Context of True and Artifactual Somatic Variants

(A and B) Clonality as defined by ABSOLUTE (A) and mappability of somatic variants (B) identified by the matched-normal WES pipeline. In each panel, the first two sets of bars enumerate the putative somatic variants identified as concordant or discordant in the technical replicates, whereas the bottom four sets of bars enumerate the somatic variants identified in intratumor biopsies and subsequently validated by high-depth amplicon sequencing. High-mappability regions are regions with mappability score of 1 (see Supplemental Experimental Procedures).

(C) Comparison of the mutational spectra of validated somatic heterogeneous mutations and artifactual somatic mutations that failed validation in all samples. The reference base listed (C or T) includes the corresponding reverse complement (G or A).

(D) Distribution of signature weights obtained from the decomposition of mutational signatures from each tumor sample.

(E) Detailed mutational spectra of the trinucleotide context of the pool of mutations detected in all tumor samples. Trinucleotide contexts with significant enrichment in the validated somatic heterogeneous mutations or in the artifactual somatic mutations are shown with an asterisk above the corresponding bars. *$p < 0.005$. Statistical comparisons in (A), (B), (C), and (E) are based on Fisher's exact tests. Statistical comparisons in (D) are based on Wilcoxon tests. All statistical tests are two-sided. A p value < 0.05 was considered to indicate statistical significance. See also Figures S4–S6.

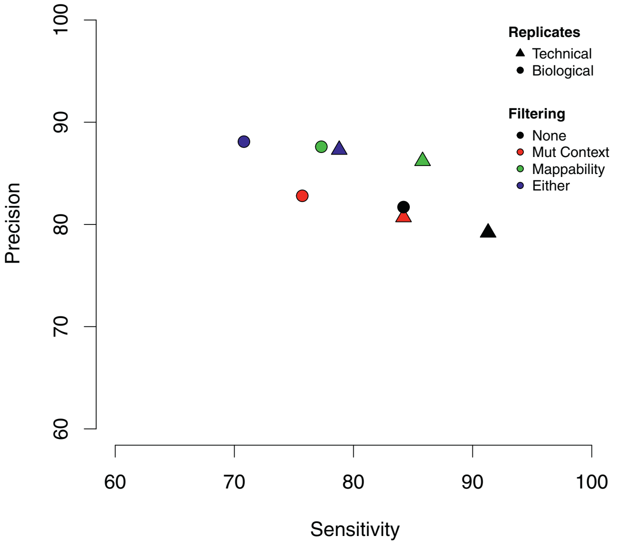

Figure 5. Effect of Filtering of Variants Called by WES Pipelines in Technical Replicates and Intratumor Biopsies

Excluding somatic mutations in low-mappability regions improves precision (or positive predictive value) by reducing false-positive calls, while slightly reducing the sensitivity by increased false-negative calls.

DISCUSSION

WES is an appealing and increasingly affordable technology to study the extent of ITGH in an unbiased manner (i.e., without *a priori* selecting genes of interest for sequencing). The reliability of WES to detect low-frequency mutations, which often account for the majority of ITGH within a cancer, and therefore to resolve clonal architecture (Figure S6) depends on the experimental design, the sequencing depth, tumor purity, and the bioinformatics approaches used to define the somatic variants. To examine the influence of these factors on measuring ITGH, here we generated a dataset incorporating both technical and biological replicates sequenced to depths commonly found in clinical or translational research studies, and validated every putative somatic variant detected with orthogonal high-depth sequencing methods. All datasets generated in this study have been made publicly available to provide a resource for the community to refine analytical tools for ITGH detection from WES.

We performed six pairs of technical replicates that involved independent library preparation and sequencing of aliquots from the same DNA extractions, and experiments were performed on different days. The technical replicates revealed an unexpectedly high degree of discordance in the putative somatic variants identified, even using the current best practice matched-normal variant calling analysis approach. Subsequent validation with high-depth amplicon sequencing (605× median coverage) of all variants identified by WES demonstrated that the majority of the false-positive somatic variants (1) displayed low VAF and were often detected as subclonal in one experiment but not in the other, (2) were in fact germline-like variants that appeared as heterogeneous somatic mutations (Kim and Speed, 2013), or (3) map to genomic regions of low mappability. The enrichment of subclonal mutations among the discordant mutations in the WES technical replicates is expected, given the well-known difficulty in identifying somatic mutations at low VAF. Indeed, comparing the putative mutations that did not validate to the "true" somatic mutations validated by high-depth amplicon sequencing demonstrated that mutations with low VAF and/or low alternative coverage were more difficult to be reliably identified by WES. On the other hand, our results revealed a not insignificant proportion of germline-like false-positive mutation calls. Although some of these germline-like variants are genuinely germline alleles not detected in the matched normal samples, a substantial proportion of these are likely attributed to alignment and sequencing biases (Kim and Speed, 2013) that manifested as false-positive variants at low VAF or alternative coverage. Importantly, our results highlighted the often overlooked importance of adequate coverage for the matched-normal sample in the accurate identification of somatic mutations, given that mutations that failed validation were, on average, associated with lower coverage in the normal sample. In terms of mappability of genomic regions, we found that false-positive mutation calls were enriched in genomic regions of poor mappability. In fact, we demonstrated that this may represent a reasonable filter if specificity is of paramount importance and some trade-off in sensitivity can be tolerated. Although our study provides direct evidence in support of ITGH, the mutations implicated in ITGH showed substantial overlap with the alterations stemming from intrinsic technical noise in terms of VAF, alternate allele depth, total depth in tumor and normal, as well as mappability. Incorporating unique molecular identifiers into deep sequencing experiments will likely reduce false positives and enhance sensitivity in detecting subclonal mutations with greater confidence (Salk et al., 2018). Furthermore, because private mutations are less likely to be identified in biopsies of relatively low purity, ITGH should be best assessed in biopsies with uniformly high cellularity.

Our analysis of the mutational signatures between the validated mutations implicated in ITGH versus the false-positive mutations revealed striking differences. The heterogeneous mutations were enriched in a pattern typically associated with increased APOBEC activity, and this pattern has been previously shown to contribute to ITGH in breast and lung cancers (de Bruin et al., 2014; Ng et al., 2017). On the other hand, the false-positive mutations were enriched for, in particular, C > A and T > G mutations at specific sequence contexts. A recent study of rare polymorphisms determined by high-depth whole-genome sequencing in 300 individuals of diverse genetic origins identified four mutational signatures, two of which were consistent within populations and had a clear association with geographic distribution (Mathieson and Reich, 2017). The origin of the remaining two were uninterpretable, with one of these latter signatures dominated by T > G mutations in the GpTpG context and the other signature highly correlated with COSMIC signature 5, which has been found in all cancer types (Alexandrov et al., 2013) and has been suggested to display clock-like properties suggestive of an association with the aging process (Alexandrov et al., 2015). Our

analysis of the sequence context of false-positive variants identified both these features as being significantly enriched in artifactual putative mutations (Figures 4C–4E), strongly suggesting that caution should be exercised in the interpretation of the reported mutational signatures.

This study has several limitations. The sample size of 6 breast cancers with 18 biopsies may be too limited to allow generalizing our results on ITGH to all breast cancer subtypes or to other cancers. Of note, the unique nested experimental design incorporating within-sample technical replication, processed and sequenced in the same manner as the intratumor biopsies, provided an estimate of background discordance against which the ITGH results could be interpreted. Additionally, the extensive orthogonal validation by high-depth amplicon sequencing on an independent sequencing platform with very different chemistry from the platform used for WES adds rigor to our study. The sequencing depth attained in this study is comparable with that in previous studies using WES (with subsequent high-depth sequencing for validation) for the genomic characterization of ITGH (Yates et al., 2015); it is plausible, however, that WES at higher depth (i.e., >250×) would mitigate in part the false positives and false negatives, in particular in samples with tumor cell content < 50%. Finally, the three WES analyses pipelines used different calling algorithms and filtering steps that reflected the best practice at the time of the analysis, but future improvements could result in reduced bias.

In summary, our study showed that WES at 184 mean depth of coverage in the tumor samples overestimates the extent of ITGH, and the technical noise associated with somatic mutation detection using WES alone can confound true ITGH. Our results also suggested that it is not possible to reduce the false-positive rate through more aggressive minimum depth filtering without affecting the sensitivity of detecting true somatic mutations in the 1%–5% VAF range, but excluding mutations that occur in low-mappability regions of the genome, or in certain mutational contexts could reduce artifactual somatic mutations and provide less biased estimates of ITGH. Nevertheless, orthogonal, high-depth validation experiments are highly desirable in the context of quantifying ITGH.

EXPERIMENTAL PROCEDURES

Tumor Sample Collection

Breast cancer samples were collected from patients with newly diagnosed invasive breast cancer with tumor size > 2 cm at the Yale Cancer Center. Tumor tissues were obtained with three punch biopsies at least 1 cm apart from three different regions of the tumor after pathologic gross examination. Six of these tumors with high enough cellularity (>50%) and high DNA quality from all three biopsies and with matched blood DNA were selected for this study.

WES and Analysis

DNA was extracted and library was prepared using standard protocols, and the exome was captured using the NimbleGen SeqCap EZ Human Exome Kit version 2.0. Sequencing was performed on the HiSeq 2000 in paired-end 75-cycle mode at the Yale Center for Genome Analysis. We used three different analytical pipelines for detecting variants. A single-sample "tumor-only" pipeline, a "cohort-normal" pipeline using an in-house normal reference obtained from ten unrelated normal blood DNA samples, and a "matched-normal" pipeline using the matched-normal DNA from each patient as reference. Further details are provided in Supplemental Experimental Procedures.

Validation of Putative Variants with High-Depth Amplicon Sequencing

Variants identified by WES were subjected to validation with high-depth amplicon sequencing using custom AmpliSeq panels on the same tumor and matched normal DNA samples. Amplicon sequencing was performed to a median depth of 600×. Further details are provided in Supplemental Experimental Procedures.

Mutational Signature Analysis and Mappability

Mutational signature analyses comparing the validated variants that contribute to ITHG and the WES false-positive calls were performed for individual tumor samples and for the pooled mutations over all samples using the R package deconstructSigs (Rosenthal et al., 2016). Mappability of SNVs was assessed using the CRG Alignability track (Derrien et al., 2012) in the UCSC Genome Browser. Additional details are provided in Supplemental Experimental Procedures.

DATA AND SOFTWARE AVAILABILITY

The accession number for the raw data from WES and AmpliSeq sequences reported in this paper is SRA: SRP070662.

SUPPLEMENTAL INFORMATION

Supplemental Information includes Supplemental Experimental Procedures, six figures, and four tables and can be found with this article online at https://doi.org/10.1016/j.celrep.2018.10.046.

ACKNOWLEDGMENTS

Research reported in this publication was supported in part by the Breast Cancer Research Foundation (to L.P., J.S.R.-F., and C.H.) and by a Cancer Center Support Grant of the NIH/National Cancer Institute (grant P30CA008748). S.P. is currently funded by the Swiss National Foundation (Ambizione grant PZ00P3_168165). The content is solely the responsibility of the authors and does not necessarily represent the official views of the NIH. The funders had no role in study design, data collection and analysis, decision to publish, or preparation of the manuscript.

AUTHOR CONTRIBUTIONS

Conceptualization, B.W., L.P., J.S.R.-F., and C.H.; Methodology, W.S., C.K.Y.N., M.B.G., L.P., J.S.R.-F., and C.H.; Software, W.S., C.K.Y.N., R.S.L., T.J., S.K., and X.L.; Validation, C.K.Y.N., R.S.L., B.W., and J.S.R.-F.; Formal Analysis, W.S., C.K.Y.N., R.S.L., T.J., S.P., and C.H.; Investigation, V.B.W., A.B.C., and B.W.; Resources, A.B.C., M.B.G., B.W., L.P., J.S.R.-F., and C.H.; Data Curation, V.B.W. and A.B.C.; Writing – Original Draft, W.S., C.K.Y.N., R.S.L., B.W., J.S.R.-F. and C.H.; Writing – Review and Editing, all authors; Visualization, W.S., C.K.Y.N., R.S.L., X.L., and C.H.; Supervision, M.B.G., B.W., L.P., J.S.R.-F., and C.H.; Funding Acquisition, L.P., J.S.R.-F., and C.H.

DECLARATION OF INTERESTS

The authors declare no competing interests.

Received: January 16, 2018
Revised: May 20, 2018
Accepted: October 11, 2018
Published: November 6, 2018

REFERENCES

Alexandrov, L.B., Nik-Zainal, S., Wedge, D.C., Aparicio, S.A., Behjati, S., Biankin, A.V., Bignell, G.R., Bolli, N., Borg, A., Børresen-Dale, A.L., et al.; Australian Pancreatic Cancer Genome Initiative; ICGC Breast Cancer Consortium; ICGC

MMML-Seq Consortium; ICGC PedBrain (2013). Signatures of mutational processes in human cancer. Nature *500*, 415–421.

Alexandrov, L.B., Jones, P.H., Wedge, D.C., Sale, J.E., Campbell, P.J., Nik-Zainal, S., and Stratton, M.R. (2015). Clock-like mutational processes in human somatic cells. Nat. Genet. *47*, 1402–1407.

Carter, S.L., Cibulskis, K., Helman, E., McKenna, A., Shen, H., Zack, T., Laird, P.W., Onofrio, R.C., Winckler, W., Weir, B.A., et al. (2012). Absolute quantification of somatic DNA alterations in human cancer. Nat. Biotechnol. *30*, 413–421.

Chen, G., Mosier, S., Gocke, C.D., Lin, M.-T., and Eshleman, J.R. (2014). Cytosine deamination is a major cause of baseline noise in next-generation sequencing. Mol. Diagn. Ther. *18*, 587–593.

Cibulskis, K., Lawrence, M.S., Carter, S.L., Sivachenko, A., Jaffe, D., Sougnez, C., Gabriel, S., Meyerson, M., Lander, E.S., and Getz, G. (2013). Sensitive detection of somatic point mutations in impure and heterogeneous cancer samples. Nat. Biotechnol. *31*, 213–219.

Clark, M.J., Chen, R., Lam, H.Y.K., Karczewski, K.J., Chen, R., Euskirchen, G., Butte, A.J., and Snyder, M. (2011). Performance comparison of exome DNA sequencing technologies. Nat. Biotechnol. *29*, 908–914.

de Bruin, E.C., McGranahan, N., Mitter, R., Salm, M., Wedge, D.C., Yates, L., Jamal-Hanjani, M., Shafi, S., Murugaesu, N., Rowan, A.J., et al. (2014). Spatial and temporal diversity in genomic instability processes defines lung cancer evolution. Science *346*, 251–256.

Derrien, T., Estellé, J., Marco Sola, S., Knowles, D.G., Raineri, E., Guigó, R., and Ribeca, P. (2012). Fast computation and applications of genome mappability. PLoS ONE *7*, e30377.

Ding, L., Ellis, M.J., Li, S., Larson, D.E., Chen, K., Wallis, J.W., Harris, C.C., McLellan, M.D., Fulton, R.S., Fulton, L.L., et al. (2010). Genome remodelling in a basal-like breast cancer metastasis and xenograft. Nature *464*, 999–1005.

Do, H., and Dobrovic, A. (2015). Sequence artifacts in DNA from formalin-fixed tissues: causes and strategies for minimization. Clin. Chem. *61*, 64–71.

Fisher, R., Pusztai, L., and Swanton, C. (2013). Cancer heterogeneity: implications for targeted therapeutics. Br. J. Cancer *108*, 479–485.

Gerlinger, M., Rowan, A.J., Horswell, S., Math, M., Larkin, J., Endesfelder, D., Gronroos, E., Martinez, P., Matthews, N., Stewart, A., et al. (2012). Intratumor heterogeneity and branched evolution revealed by multiregion sequencing. N. Engl. J. Med. *366*, 883–892.

Graw, S., Meier, R., Minn, K., Bloomer, C., Godwin, A.K., Fridley, B., Vlad, A., Beyerlein, P., and Chien, J. (2015). Robust gene expression and mutation analyses of RNA-sequencing of formalin-fixed diagnostic tumor samples. Sci. Rep. *5*, 12335.

Hiley, C., de Bruin, E.C., McGranahan, N., and Swanton, C. (2014). Deciphering intratumor heterogeneity and temporal acquisition of driver events to refine precision medicine. Genome Biol. *15*, 453.

Hou, Y., Song, L., Zhu, P., Zhang, B., Tao, Y., Xu, X., Li, F., Wu, K., Liang, J., Shao, D., et al. (2012). Single-cell exome sequencing and monoclonal evolution of a JAK2-negative myeloproliferative neoplasm. Cell *148*, 873–885.

Jiang, T., Shi, W., Natowicz, R., Ononye, S.N., Wali, V.B., Kluger, Y., Pusztai, L., and Hatzis, C. (2014). Statistical measures of transcriptional diversity capture genomic heterogeneity of cancer. BMC Genomics *15*, 876.

Kim, S.Y., and Speed, T.P. (2013). Comparing somatic mutation-callers: beyond Venn diagrams. BMC Bioinformatics *14*, 189.

Koboldt, D.C., Zhang, Q., Larson, D.E., Shen, D., McLellan, M.D., Lin, L., Miller, C.A., Mardis, E.R., Ding, L., and Wilson, R.K. (2012). VarScan 2: somatic mutation and copy number alteration discovery in cancer by exome sequencing. Genome Res. *22*, 568–576.

Li, H. (2014). Toward better understanding of artifacts in variant calling from high-coverage samples. Bioinformatics *30*, 2843–2851.

Martelotto, L.G., Baslan, T., Kendall, J., Geyer, F.C., Burke, K.A., Spraggon, L., Piscuoglio, S., Chadalavada, K., Nanjangud, G., Ng, C.K.Y., et al. (2017). Whole-genome single-cell copy number profiling from formalin-fixed paraffin-embedded samples. Nat. Med. *23*, 376–385.

Marusyk, A., Almendro, V., and Polyak, K. (2012). Intra-tumour heterogeneity: a looking glass for cancer? Nat. Rev. Cancer *12*, 323–334.

Mathieson, I., and Reich, D. (2017). Differences in the rare variant spectrum among human populations. PLoS Genet. *13*, e1006581.

Morris, L.G.T., Riaz, N., Desrichard, A., Şenbabaoğlu, Y., Hakimi, A.A., Makarov, V., Reis-Filho, J.S., and Chan, T.A. (2016). Pan-cancer analysis of intratumor heterogeneity as a prognostic determinant of survival. Oncotarget *7*, 10051–10063.

Nakamura, K., Oshima, T., Morimoto, T., Ikeda, S., Yoshikawa, H., Shiwa, Y., Ishikawa, S., Linak, M.C., Hirai, A., Takahashi, H., et al. (2011). Sequence-specific error profile of Illumina sequencers. Nucleic Acids Res. *39*, e90.

Navin, N., Kendall, J., Troge, J., Andrews, P., Rodgers, L., McIndoo, J., Cook, K., Stepansky, A., Levy, D., Esposito, D., et al. (2011). Tumour evolution inferred by single-cell sequencing. Nature *472*, 90–94.

Newburger, D.E., Kashef-Haghighi, D., Weng, Z., Salari, R., Sweeney, R.T., Brunner, A.L., Zhu, S.X., Guo, X., Varma, S., Troxell, M.L., et al. (2013). Genome evolution during progression to breast cancer. Genome Res. *23*, 1097–1108.

Ng, C.K.Y., Bidard, F.-C., Piscuoglio, S., Geyer, F.C., Lim, R.S., de Bruijn, I., Shen, R., Pareja, F., Berman, S.H., Wang, L., et al. (2017). Genetic heterogeneity in therapy-naïve synchronous primary breast cancers and their metastases. Clin. Cancer Res. *23*, 4402–4415.

Nik-Zainal, S., Van Loo, P., Wedge, D.C., Alexandrov, L.B., Greenman, C.D., Lau, K.W., Raine, K., Jones, D., Marshall, J., Ramakrishna, M., et al.; Breast Cancer Working Group of the International Cancer Genome Consortium (2012). The life history of 21 breast cancers. Cell *149*, 994–1007.

Nik-Zainal, S., Davies, H., Staaf, J., Ramakrishna, M., Glodzik, D., Zou, X., Martincorena, I., Alexandrov, L.B., Martin, S., Wedge, D.C., et al. (2016). Landscape of somatic mutations in 560 breast cancer whole-genome sequences. Nature *534*, 47–54.

Qi, Y., Liu, X., Liu, C.-G., Wang, B., Hess, K.R., Symmans, W.F., Shi, W., and Pusztai, L. (2015). Reproducibility of variant calls in replicate next generation sequencing experiments. PLoS ONE *10*, e0119230.

Rosenthal, R., McGranahan, N., Herrero, J., Taylor, B.S., and Swanton, C. (2016). DeconstructSigs: delineating mutational processes in single tumors distinguishes DNA repair deficiencies and patterns of carcinoma evolution. Genome Biol. *17*, 31.

Salk, J.J., Schmitt, M.W., and Loeb, L.A. (2018). Enhancing the accuracy of next-generation sequencing for detecting rare and subclonal mutations. Nat. Rev. Genet. *19*, 269–285.

Saunders, C.T., Wong, W.S.W., Swamy, S., Becq, J., Murray, L.J., and Cheetham, R.K. (2012). Strelka: accurate somatic small-variant calling from sequenced tumor-normal sample pairs. Bioinformatics *28*, 1811–1817.

Shen, R., and Seshan, V.E. (2016). FACETS: allele-specific copy number and clonal heterogeneity analysis tool for high-throughput DNA sequencing. Nucleic Acids Res. *44*, e131.

Shi, W., Jiang, T., Nuciforo, P., Hatzis, C., Holmes, E., Harbeck, N., Sotiriou, C., Peña, L., Loi, S., Rosa, D.D., et al. (2017). Pathway level alterations rather than mutations in single genes predict response to HER2-targeted therapies in the neo-ALTTO trial. Ann. Oncol. *28*, 128–135.

Sims, D., Sudbery, I., Ilott, N.E., Heger, A., and Ponting, C.P. (2014). Sequencing depth and coverage: key considerations in genomic analyses. Nat. Rev. Genet. *15*, 121–132.

Smith, E.N., Jepsen, K., Khosroheidari, M., Rassenti, L.Z., D'Antonio, M., Ghia, E.M., Carson, D.A., Jamieson, C.H., Kipps, T.J., and Frazer, K.A. (2014). Biased estimates of clonal evolution and subclonal heterogeneity can arise from PCR duplicates in deep sequencing experiments. Genome Biol. *15*, 420.

Turner, N.C., and Reis-Filho, J.S. (2012). Genetic heterogeneity and cancer drug resistance. Lancet Oncol. *13*, e178–e185.

von Wahlde, M.-K., Timms, K.M., Chagpar, A., Wali, V.B., Jiang, T., Bossuyt, V., Saglam, O., Reid, J., Gutin, A., Neff, C., et al. (2017). Intratumor heterogeneity of

homologous recombination deficiency in primary breast cancer. Clin. Cancer Res. *23*, 1193–1199.

Wang, Y., Waters, J., Leung, M.L., Unruh, A., Roh, W., Shi, X., Chen, K., Scheet, P., Vattathil, S., Liang, H., et al. (2014). Clonal evolution in breast cancer revealed by single nucleus genome sequencing. Nature *512*, 155–160.

Wilm, A., Aw, P.P.K., Bertrand, D., Yeo, G.H.T., Ong, S.H., Wong, C.H., Khor, C.C., Petric, R., Hibberd, M.L., and Nagarajan, N. (2012). LoFreq: a sequence-quality aware, ultra-sensitive variant caller for uncovering cell-population heterogeneity from high-throughput sequencing datasets. Nucleic Acids Res. *40*, 11189–11201.

Xu, X., Hou, Y., Yin, X., Bao, L., Tang, A., Song, L., Li, F., Tsang, S., Wu, K., Wu, H., et al. (2012). Single-cell exome sequencing reveals single-nucleotide mutation characteristics of a kidney tumor. Cell *148*, 886–895.

Yap, T.A., Gerlinger, M., Futreal, P.A., Pusztai, L., and Swanton, C. (2012). Intratumor heterogeneity: seeing the wood for the trees. Sci. Transl. Med. *4*, 127ps10.

Yates, L.R., Gerstung, M., Knappskog, S., Desmedt, C., Gundem, G., Van Loo, P., Aas, T., Alexandrov, L.B., Larsimont, D., Davies, H., et al. (2015). Subclonal diversification of primary breast cancer revealed by multiregion sequencing. Nat. Med. *21*, 751–759.

Cell Reports, Volume *25*

Supplemental Information

Reliability of Whole-Exome Sequencing

for Assessing Intratumor Genetic Heterogeneity

Weiwei Shi, Charlotte K.Y. Ng, Raymond S. Lim, Tingting Jiang, Sushant Kumar, Xiaotong Li, Vikram B. Wali, Salvatore Piscuoglio, Mark B. Gerstein, Anees B. Chagpar, Britta Weigelt, Lajos Pusztai, Jorge S. Reis-Filho, and Christos Hatzis

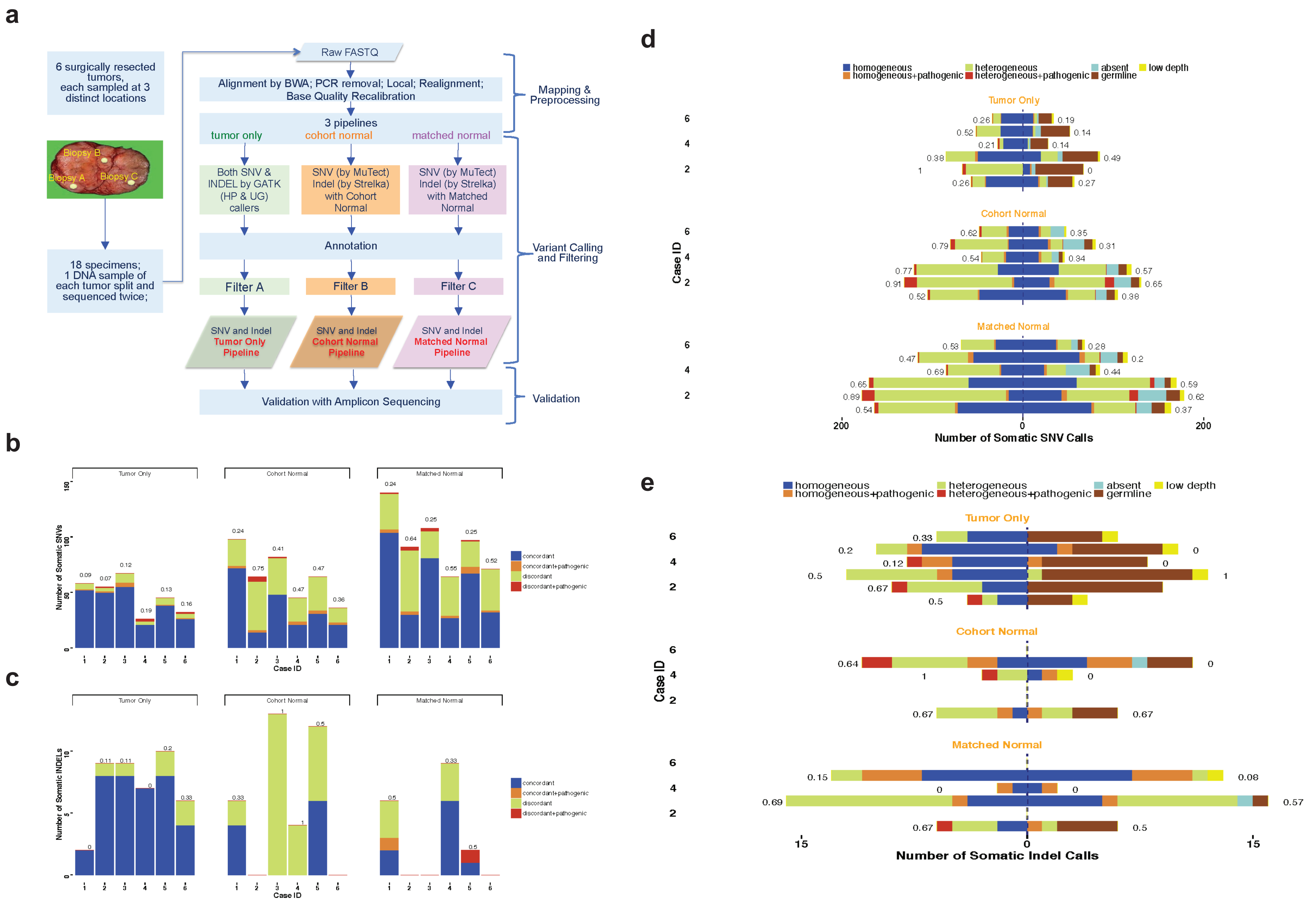
a
6 surgically resected tumors, each sampled at 3 distinct locations
Biopsy A
Biopsy B
Biopsy C
18 specimens; 1 DNA sample of each tumor split and sequenced twice;
Raw FASTQ
Alignment by BWA; PCR removal; Local; Realignment; Base Quality Recalibration
3 pipelines
tumor only
cohort normal
matched normal
Both SNV & INDEL by GATK (HP & UG) callers
SNV (by MuTect) Indel (by Strelka) with Cohort Normal
SNV (by MuTect) Indel (by Strelka) with Matched Normal
Annotation
Filter A
Filter B
Filter C
SNV and Indel Tumor Only Pipeline
SNV and Indel Cohort Normal Pipeline
SNV and Indel Matched Normal Pipeline
Validation with Amplicon Sequencing
Mapping & Preprocessing
Variant Calling and Filtering
Validation
b
Tumor Only
Cohort Normal
Matched Normal
Number of Somatic SNVs
Case ID
concordant
concordant+pathogenic
discordant
discordant+pathogenic
c
Number of Somatic INDELs
Case ID
d
homogeneous
homogeneous+pathogenic
heterogeneous
heterogeneous+pathogenic
absent
germline
low depth
Case ID
Number of Somatic SNV Calls
e
Case ID
Number of Somatic Indel Calls

Figure S1. (a) Experimental design and analytical pipelines for calling somatic variants, Related to Figure 1. DNA from three intra-tumor biopsies plus an additional technical replicate from each of six breast tumors was sequenced using whole exome sequencing. Raw DNA reads were processed and aligned to the hg19 or the GRCh37 human reference genome using BWA. Subsequently, somatic SNVs and INDELs were identified by three independent pipelines: 1) tumor-only using only the tumor DNA, 2) cohort-normal using the tumor DNA and a reference germline DNA pooled from ten unrelated blood DNA samples, and 3) matched-normal using the tumor DNA and blood DNA from the same patients. Each pipeline used different filters to exclude false positive somatic variants as described in the **Supplemental Experimental Procedures**. The identified somatic variants were subsequently validated by high-depth amplicon sequencing. Further details are provided in **Supplemental Experimental Procedures**.

(b-c) Comparison of technical variation in somatic variants identified from the three WES pipelines, Related to Figure 1; Figure 2. Comparison of the number of somatic **(b)** SNVs and **(c)** INDELs identified in the technical replicate pairs from each tumor using the tumor-only, cohort- normal, and matched-normal WES pipelines. Each bar corresponds to a technical replicate. The x-axis of these graphs is the total number of somatic variants identified by each pipeline in the pair of technical replicates from each tumor. The number above each bar is the Jaccard distance for the set of variants identified in each pair of technical replicates from a given tumor, which is a measure of the technical variation of the pipelines.

(d-e) Intratumor heterogeneity in somatic SNVs and INDELS detected by whole exome sequencing relative to deep sequencing, Related to Figure 1; Figure 2. Comparison of the number of somatic **(d)** SNVs and **(e)** INDELs identified in the intratumor biopsies from each tumor using the three WES pipelines (left) and their validation status according to high-depth amplicon sequencing (right). All putative WES somatic variants in the intratumor biopsies were validated by high-depth amplicon sequencing and classified as low depth (<50x), absent (VAF<1%), germline (tumor VAF<5x germline VAF), somatic and somatic (low VAF, **Table S3**). Homogeneous somatic variants were those identified in all three intratumor biopsies and heterogeneous were those detected in one or two biopsies. Pathogenicity of variants was assessed as described in **Supplemental Experimental Procedures**. The extent of intratumor heterogeneity in the somatic variant calls was quantified as the Jaccard distance for each case and is shown next to each bar.

a

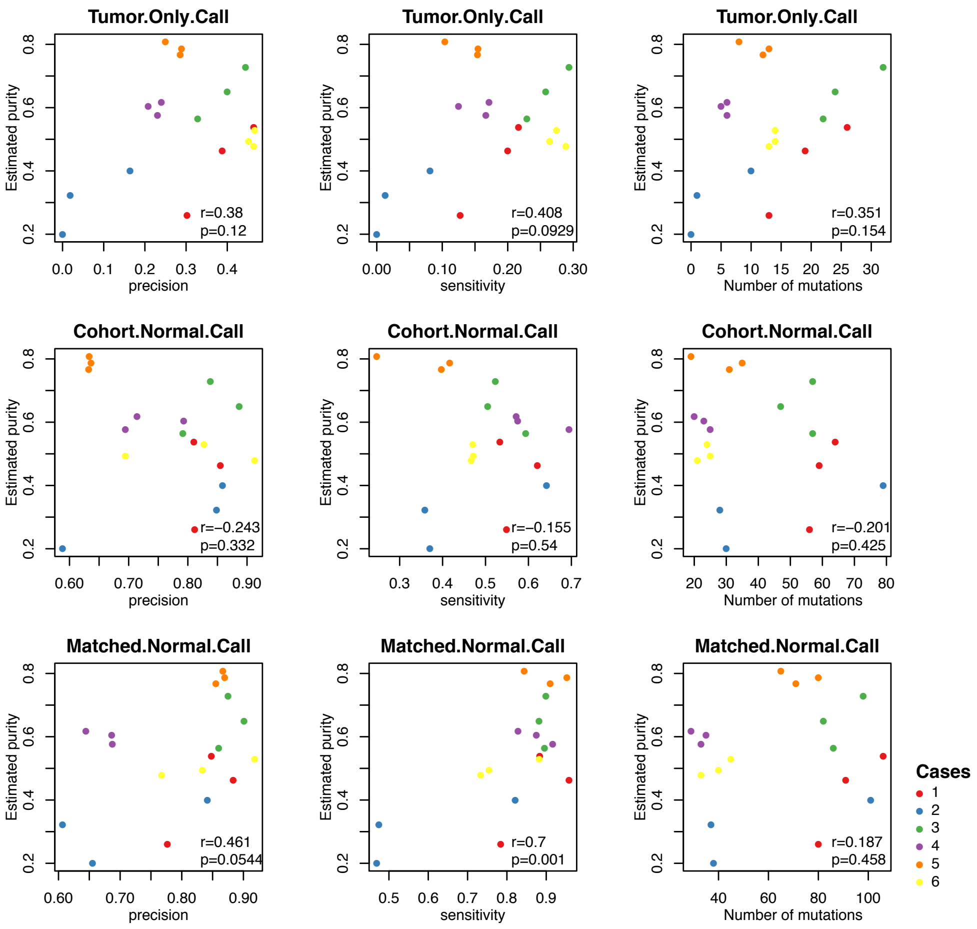

Tumor.Only.Call
Estimated purity
precision
r=0.38
p=0.12
sensitivity
r=0.408
p=0.0929
Number of mutations
r=0.351
p=0.154
Cohort.Normal.Call
r=−0.243
p=0.332
r=−0.155
p=0.54
r=−0.201
p=0.425
Matched.Normal.Call
r=0.461
p=0.0544
r=0.7
p=0.001
r=0.187
p=0.458
Cases
1
2
3
4
5
6

b

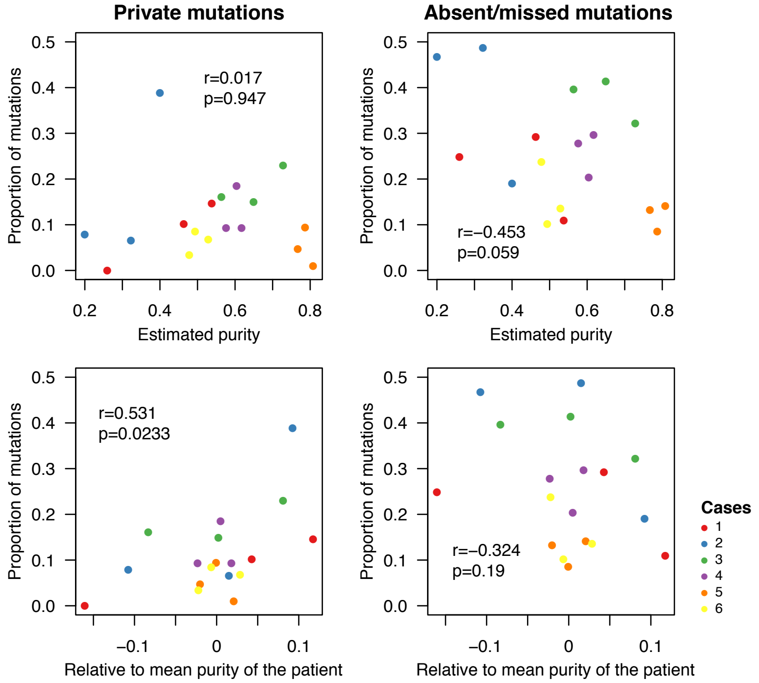

Private mutations
Absent/missed mutations
Proportion of mutations
Estimated purity
r=0.017
p=0.947
r=−0.453
p=0.059
Relative to mean purity of the patient
r=0.531
p=0.0233
r=−0.324
p=0.19
Cases
1
2
3
4
5
6

Figure S2. (a) Relationship between tumor purity and performance characteristics of the three WES analysis pipelines, Related to Figure 2. Precision and sensitivity are defined as in Fig. 2c. Each dot represents a tumor biopsy, color coded by the patients. Tumor purity was estimated using FACETS. Correlation was computed as Pearson correlation (r). The estimated purity for Case 2 biorep C, for which purity could not be estimated by FACETS, was set to 0.2.

(b) Relationship between tumor purity and intratumor genetic heterogeneity, Related to Figure 2. The proportion of private mutations (i.e. mutations found only in a given biopsy as a proportion of the union of all validated somatic mutations in a given patient, left) and absent/missed mutations (i.e. mutations found in at least one other biopsy but not the biopsy of interest) are plotted against estimated purity as defined by FACETS (top) and estimated purity relative to the mean purity of all intratumor biopsies of a given patient (bottom). Each dot represents a tumor biopsy, color coded by patients. Correlation was computed as Pearson correlation (r). The estimated purity for Case 2 biorep C, for which purity could not be estimated by FACETS, was set to 0.2.

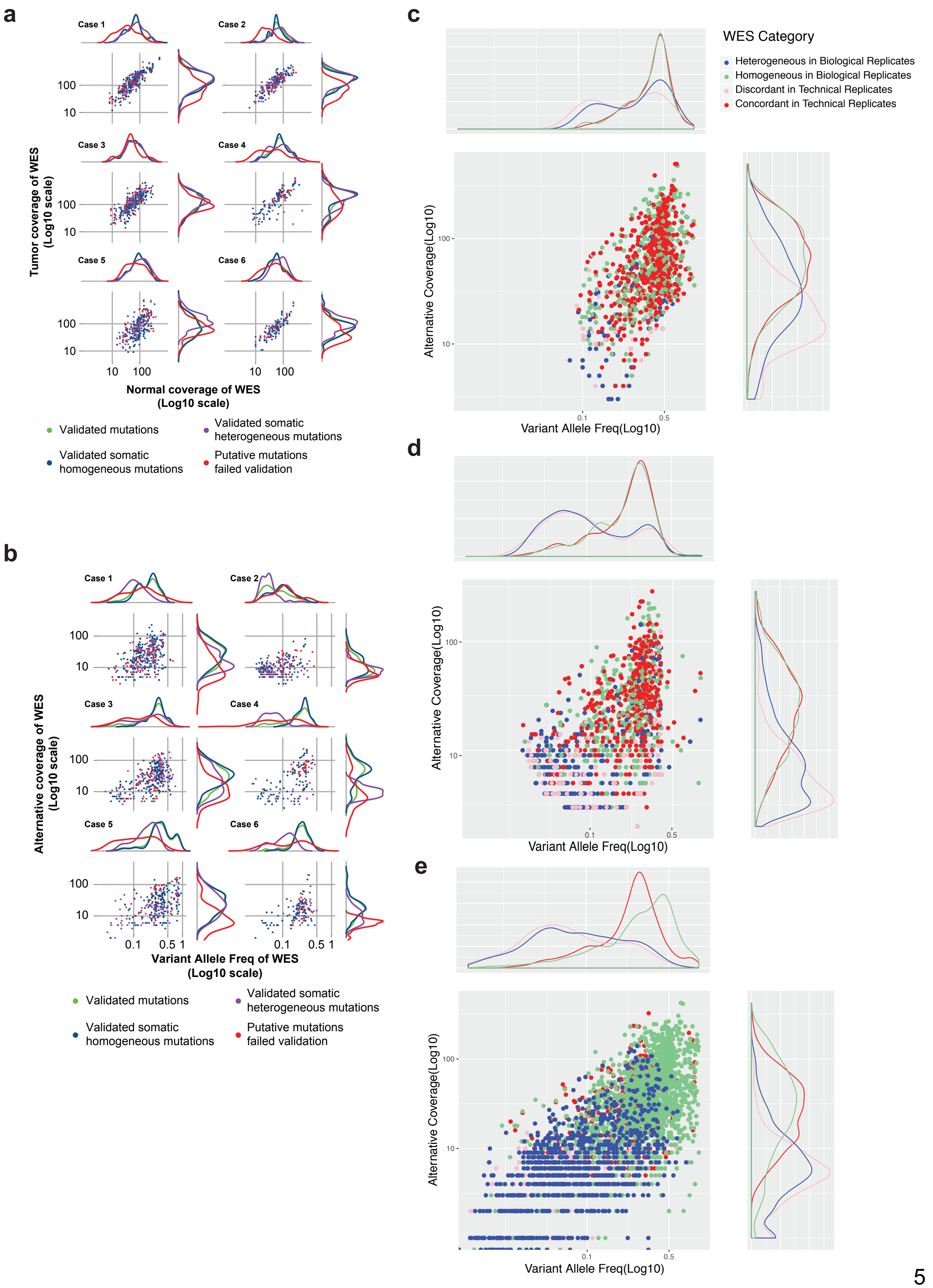
a
Case 1
Case 2
Case 3
Case 4
Case 5
Case 6
Tumor coverage of WES (Log10 scale)
Normal coverage of WES (Log10 scale)
Validated mutations
Validated somatic heterogeneous mutations
Validated somatic homogeneous mutations
Putative mutations failed validation
b
Alternative coverage of WES (Log10 scale)
Variant Allele Freq of WES (Log10 scale)
c
WES Category
Heterogeneous in Biological Replicates
Homogeneous in Biological Replicates
Discordant in Technical Replicates
Concordant in Technical Replicates
Alternative Coverage(Log10)
Variant Allele Freq(Log10)
d
e

Figure S3. (a) Coverage characteristics of true somatic variants and false positive mutations in the WES data for individual patients, using the matched-normal WES pipeline, Related to Figure 3. Total coverage in the tumor is plotted against the coverage in the matched normal sample of somatic mutations identified in all the specimens of a given patient. The validation status categories are the same as in panels Figure 3. Density kernel plots of the marginal distributions are included above and to the right of the scatter plots for each of the four categories of mutations.

(b) Coverage characteristics of true somatic variants and false positive mutations in the WES data for individual patients, using the matched-normal WES pipeline, Related to Figure 3. WES alternative allele coverage is plotted against VAF of somatic mutations identified in all the specimens of a given patient. The validation status categories are the same as in panels Figure 3. Density kernel plots of the marginal distributions are included above and to the right of the scatter plots for each of the four categories of mutations.

(c-e) Characteristics of variants called by WES pipelines in the intratumor biopsies, Related to Figure 3. Alternative allele coverage from WES versus variant allele fraction (log10 scale) for variants identified by **(c)** tumor-only, **(d)** cohort-normal or **(e)** matched-normal WES analysis pipelines in the intratumor biopsies from all cases. These calls include all putative somatic variants as defined by each of the pipelines prior to validation by amplicon sequencing. Variants are labeled as concordant in the technical replicates, discordant in the technical replicates, homogeneous if they were identified in all three biopsies, and heterogeneous if they were identified in one or two of the biopsies.

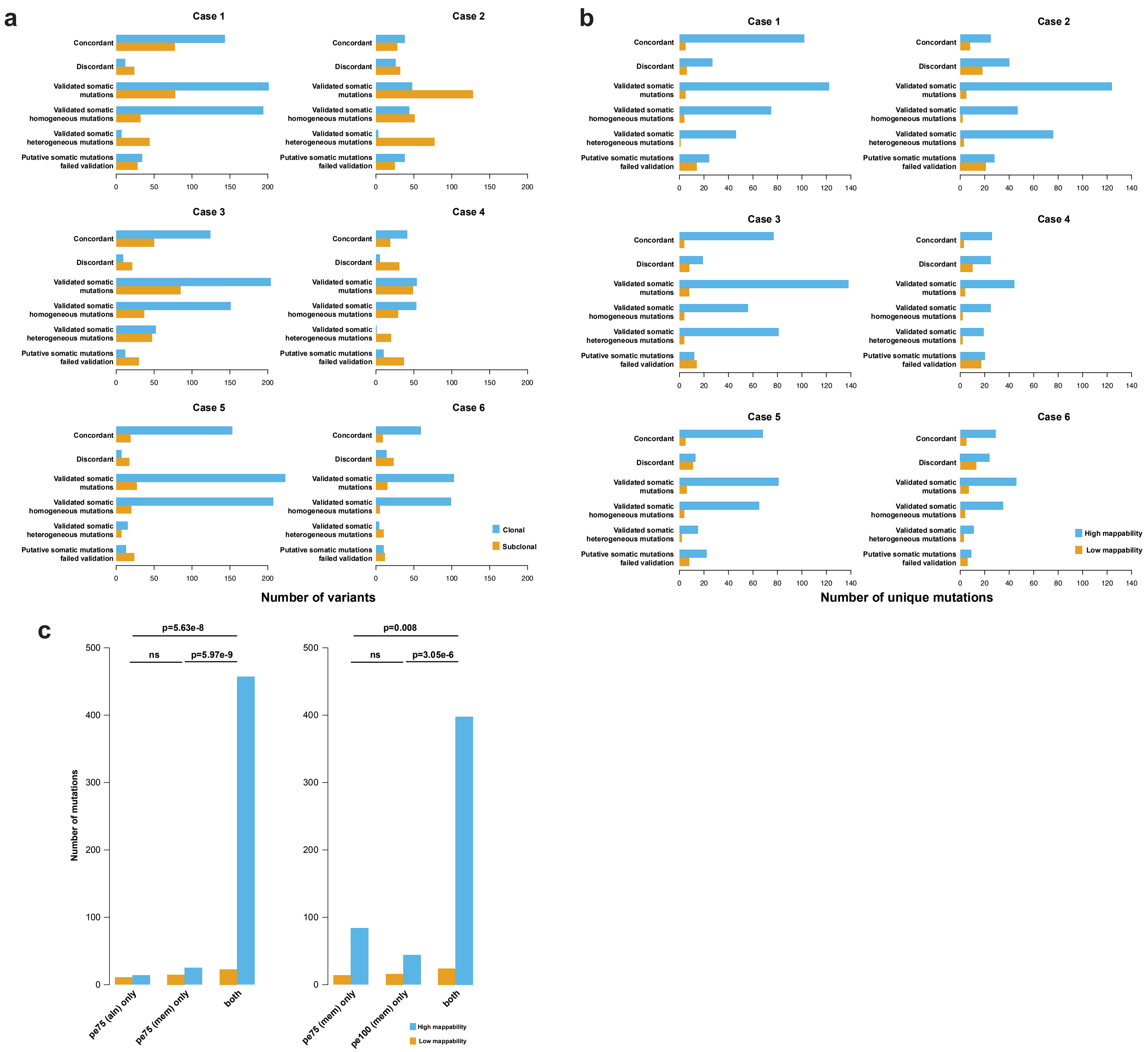
a
Case 1
Case 2
Case 3
Case 4
Case 5
Case 6
Concordant
Discordant
Validated somatic mutations
Validated somatic homogeneous mutations
Validated somatic heterogeneous mutations
Putative somatic mutations failed validation
Clonal
Subclonal
Number of variants
b
High mappability
Low mappability
Number of unique mutations
c
p=5.63e-8
ns
p=5.97e-9
p=0.008
p=3.05e-6
Number of mutations
pe75 (aln) only
pe75 (mem) only
pe100 (mem) only
both

Figure S4. (a) Clonality as defined by ABSOLUTE of true and artifactual somatic variants identified by the matched-normal WES pipeline for individual patients, Related to Figure 4. In each panel, the first two sets of bars enumerate the putative somatic variants identified as concordant or discordant in the technical replicates, whereas the bottom four sets of bars enumerate the somatic variants identified in intratumor biopsies and subsequently validated by high-depth amplicon sequencing.

(b) Mappability of true and artifactual somatic variants identified by the matched-normal WES pipeline for individual patients, Related to Figure 4. In each panel, the first two sets of bars enumerate the putative somatic variants identified as concordant or discordant in the technical replicates, whereas the bottom four sets of bars enumerate the somatic variants identified in intratumor biopsies and subsequently validated by high-depth amplicon sequencing. High mappability regions are regions with mappability score of 1.

(c) Mappability of somatic variants identified by the matched-normal WES pipeline, comparing the effects of alignment algorithms and read length, using 10 breast cancer cases from TCGA, Related to Figure 4. Comparison of mappability of somatic variants identified (left) between paired-end 75bp reads aligned with BWA aln and BWA mem and (right) between paired-end 75bp reads and paired-end 100bp reads aligned with BWA mem. Comparisons were performed using WES data from 10 tumor-normal pairs from the TCGA breast cancer cohort, originally sequenced using 100bp paired-end sequencing. To obtain 75bp reads, reads were trimmed to 75bp prior to alignment. For the 100bp reads, reads were aligned as is, then downsampled to 75% to match the overall sequencing depth of the trimmed 75bp reads. Somatic mutations were called using the matched-normal WES pipeline. Statistical comparisons were performed using Fisher exact tests.

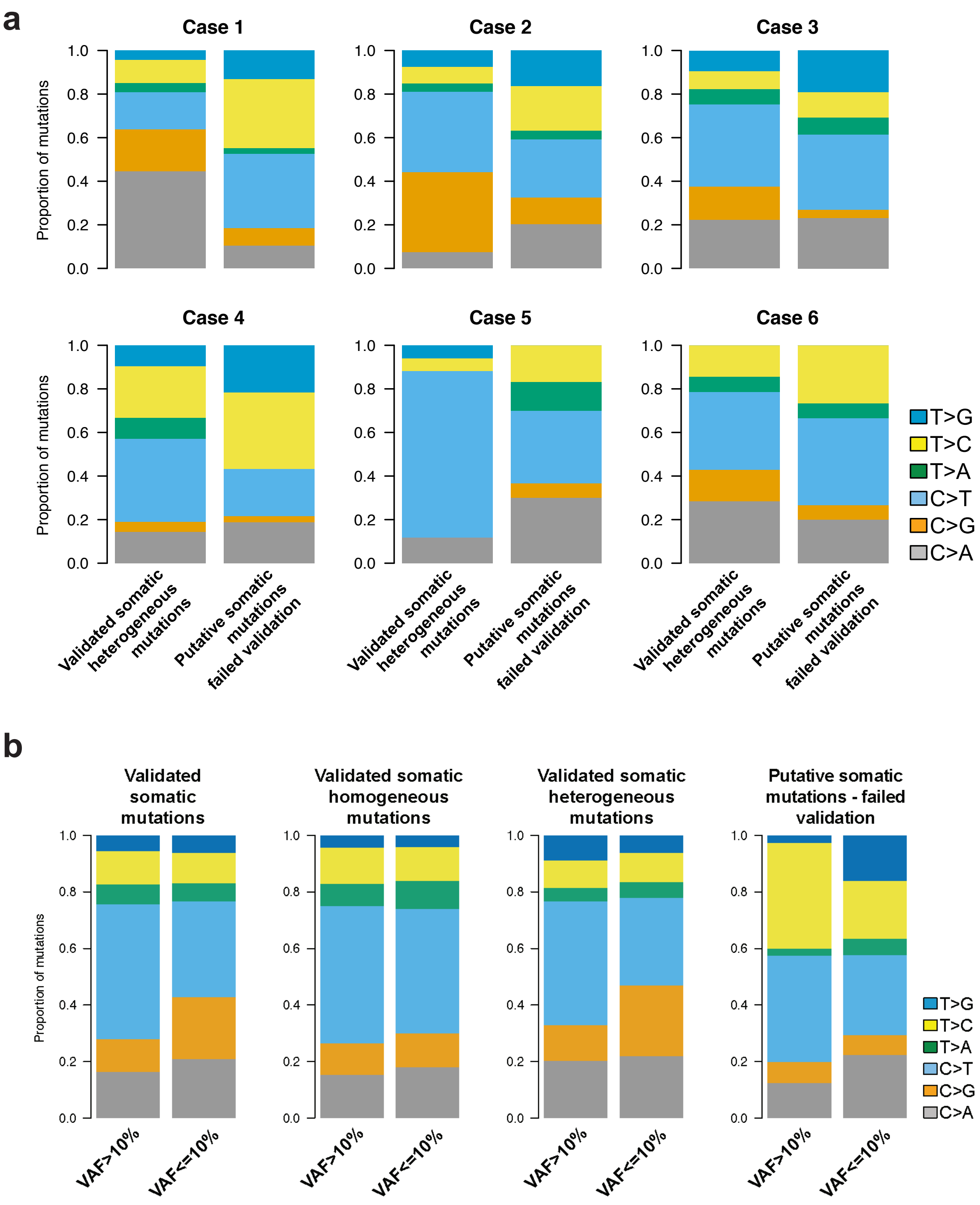
a
Case 1
Case 2
Case 3
Case 4
Case 5
Case 6
Proportion of mutations
Validated somatic heterogeneous mutations
Putative somatic mutations failed validation
T>G
T>C
T>A
C>T
C>G
C>A
b
Validated somatic mutations
Validated somatic homogeneous mutations
Validated somatic heterogeneous mutations
Putative somatic mutations - failed validation
VAF>10%
VAF<=10%

Figure S5. (a) Mutational spectra of true and artifactual somatic variants identified by the matched-normal WES pipeline for individual patients, Related to Figure 4. Comparison of the mutational spectra of validated somatic heterogenous mutations and artifactual somatic mutations that failed validation in all samples for individual patients. The reference base listed (C or T) includes the corresponding reverse complement (G or A).

(b) Mutational spectra of true and artifactual somatic variants identified by the matched-normal WES pipeline stratified by VAF, Related to Figure 4. Comparison of the mutational spectra of validated somatic, validated somatic homogeneous, validated somatic heterogenous and artifactual somatic mutations that failed validation in all samples. Mutations are stratified into VAF>10% and VAF<=10%. The reference base listed (C or T) includes the corresponding reverse complement (G or A).

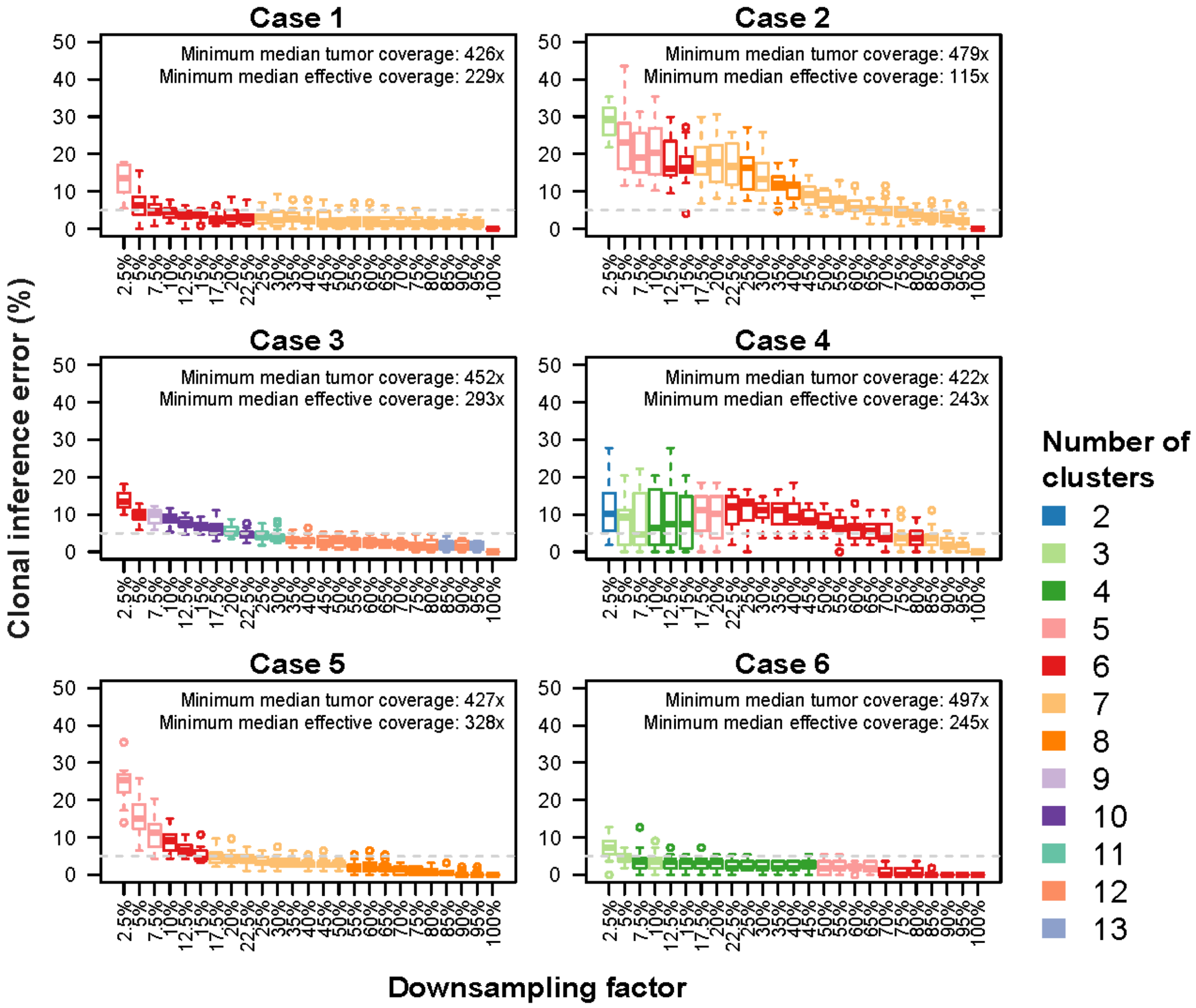
Case 1
Minimum median tumor coverage: 426x
Minimum median effective coverage: 229x
Case 2
Minimum median tumor coverage: 479x
Minimum median effective coverage: 115x
Case 3
Minimum median tumor coverage: 452x
Minimum median effective coverage: 293x
Case 4
Minimum median tumor coverage: 422x
Minimum median effective coverage: 243x
Case 5
Minimum median tumor coverage: 427x
Minimum median effective coverage: 328x
Case 6
Minimum median tumor coverage: 497x
Minimum median effective coverage: 245x
Clonal inference error (%)
Downsampling factor
Number of clusters
2
3
4
5
6
7
8
9
10
11
12
13

Figure S6. Impact of sequencing depth on clonal inference, Related to Figure 4. Boxplot showing clonal inference error at various degrees of downsampling sequencing depth. Clonal inference was performed using PyClone and clonal inference error was calculated in relation to no downsampling (i.e. 100% in the figure). Clonal inference error was calculated as 1-(the number of mutations in the same clusters as no downsampling/the total number of mutations). Downsampling was performed at 2.5% increments up to 25%, then at 5% increments, for 20 iterations. All somatic mutations were included, regardless if they would have been considered somatic at the reduced depth. Boxplots are colored according to the median number of clusters identified in the 20 runs. The minimum median tumor coverage refers to the minimum sample-level median depth of the mutations that were somatic in at least one sample in a given case (i.e. homogeneous or heterogeneous mutations). The median effective coverage refers to the sample-level median depth (as above) multiplied by its estimated tumor purity. Case 2 biorep C, for which purity could not be estimated by FACETS, was set to 0.2. The grey dotted line indicates 5% clustering error. Note the overlap between the minimum median tumor coverage and the minimum median effective coverage between the 4 cases with stable clonal inference (Cases 1, 3, 5, and 6) vs the 2 cases with unstable clonal inference (Cases 2 and 4).

SUPPLEMENTAL EXPERIMENTAL PROCEDURES

Tumor sample collection

Breast cancer samples were collected in the context of a prospective study to assess within-tumor genomic heterogeneity as previously published (Qi et al., 2015). Patients with newly diagnosed invasive breast cancer with tumor size > 2 cm were eligible and were recruited between 9th of January 2012 and 13th of November 2013 at the Yale Cancer Center. Tumor tissues were obtained with 2-3 punch biopsies at least 1 cm apart from 3 different regions of a tumor after pathologic gross examination had been completed. One biopsy from each location was formalin fixed and embedded in paraffin and the remaining biopsies were collected into RNAlater™ and stored at -80°C until DNA extraction. Tumor cellularity of each biopsy was assessed on the hematoxylin-and-eosin stained formalin-fixed sister biopsy from a given tumor location. We processed DNA for WES only if the biopsy cellularity was ≥50%. This study was approved by the Yale Cancer Center human investigations committee and all patients signed informed consent. Six tumors from this cohort with high quality DNA from all three biopsies and matched blood DNA were selected in this study to represent different breast cancer subtypes and disease spectra (**Table 1**).

Whole-exome sequencing

DNA from the three biopsies and matched blood from each of the six cases was extracted using the AllPrep Universal Kit (Qiagen). 1 µg genomic DNA was sheared to mean fragment length of 140 bp using the Covaris E210 instrument and purified by Magnetic AMPure XP beads (Beckman Coulter), and subsequently labeled with 6 base barcode during PCR amplification. The NimbleGen SeqCap EZ Human Exome Kit v2.0 (Roche) was used for exome capture following manufacturer's instructions. Intratumor biopsies and technical replicates were assigned uninformative identifiers to allow blinded sample processing, and subsequent library preparation and sequencing. Libraries were sequenced on Illumina HiSeq 2000 in paired-end 75-cycles mode at the Yale Center for Genome Analysis to a median depth of coverage of tumor samples and normal samples of 184 (range 92–211) and 90 (range 80–138), respectively **(Table S2**).

Whole-exome sequencing (WES) analysis pipelines

We used three different analytical pipelines for WES analysis (**Fig. S1a**). The single-sample "tumor-only" pipeline used only the reads derived from the tumor samples to define likely somatic mutations. Sequence reads were aligned to the human genome reference sequence version hg19 using the Burrows-Wheeler Aligner (Li and Durbin, 2009) (BWA, v0.6.2). PCR duplicates were removed using MarkDuplicates algorithm from Picard (version 1.47, http://picard.sourceforge.net/). Local realignment was performed using GATK (v3.1-1) (McKenna et al., 2010) around novel and known variant sites followed by GATK base quality recalibration. The overlap between the GATK HaplotypeCaller and UnifiedGenotyper algorithms was used to define mutations. Those identified by both callers were annotated by ANNOVAR (Yang and Wang, 2015) and non-exonic variants in regions with low mappability (Zook et al., 2014) or those outside the exome capture regions were excluded. We further excluded as putative germline SNPs those present in 1000Genomes phase1 (2014 Oct. http://www.1000genomes.org/), ESP6500 (http://evs.gs.washington.edu/EVS/), ExAC01 (http://exac.broadinstitute.org/about) or dbSNP (Build 138; variants not flagged as somatic or clinical or as having a minor allele frequency >1%).

The cohort-normal pipeline used an in-house normal reference obtained from 10 unrelated normal blood DNA samples sequenced using the same protocol, each downsampled to 20% and then pooled. Sequence reads were aligned to the human reference genome (GRCh37) using the Burrows-Wheeler Aligner (BWA, v0.6.2), followed by PCR duplicate removal, local realignment and base quality recalibration as described above. Somatic SNVs and small somatic insertions and deletions (INDELs) were defined by MuTect (Cibulskis et al., 2013) (v.1.1.4) and Strelka (Saunders et al., 2012) (v.1.0.14), respectively, using the pool of normal reads as reference and annotated by ANNOVAR (Yang and Wang, 2015). Recurrent variants with five or more occurrences in COSMIC (v.64) or ClinVar (http://www.ncbi.nlm.nih.gov/clinvar/) were whitelisted. Variants outside the target region were excluded. Low confidence somatic calls, defined as those with total coverage of fewer than 15 reads in the tumor, were present in at least five normal breast samples from the TCGA cohort (Koboldt et al., 2012a), considered likely germline in dbSNP (Build 138), or were present in ESP6500, 1000Genomes or ExAC01 were also excluded. We further removed variants with tumor variant allele fraction (VAF) < 5 times of that in the pooled normal DNA or with tumor VAF between 0.45 and 0.55. All remaining variants were manually inspected in the Integrative Genomics Viewer (Robinson et al.,

2011).

The “matched-normal” pipeline is considered the best-practice pipeline for identifying somatic events. Sequence alignment and processing up to base quality recalibration are the same as for the cohort-normal pipeline. Somatic SNVs and INDELs were detected using the sequencing reads derived from matched normal DNA from each patient as the reference. Somatic SNVs were defined using MuTect (Cibulskis et al., 2013); small INDELs were identified by the intersection of Strelka (Saunders et al., 2012) and VarScan 2 (Koboldt et al., 2012b), and further curated by manual inspection. Only variants at positions with total read depth >5 in both the tumor and normal were considered. Variants outside the target region or those supported by <5 reads were disregarded (De Mattos-Arruda et al., 2014; Martelotto et al., 2015). Variants covered by <20 reads in the germline of which more than one supported the mutant allele were disregarded. Variants covered by at least 20 reads in the germline were disregarded if the VAF in the tumor was < 5 times than that of the VAF in the germline. Variants present at global minor allele frequency >1% in dbSNP (version 138) were disregarded. Variants were annotated using SnpEff (Cingolani et al, 2012).

The identification of allele-specific copy number alterations (CNAs) and the estimation of tumor cellularity were performed using FACETS (Shen and Seshan, 2016), which performs a joint segmentation of the total and allelic copy ratio and infers allele-specific copy number states, using the reads derived from tumor samples and their matched normal counterparts. Regions of loss of heterozygosity were defined as regions having lesser (minor) copy number of zero.

Assessing technical variance of tumor cellularity estimated from WES

Estimates of tumor cellularity from technical replicate biopsies were obtained from FACETS (Shen and Seshan, 2016). We used a linear mixed-effects model with fixed and random intercept terms to estimate the mean cellularity and intratumor standard deviation. The error term provided an estimate of the within tumor or technical standard deviation of estimated cellularity.

Validation of putative somatic variants with high-depth amplicon sequencing (Ampliseq)

Variants identified by WES in both the biological and the technical replicates were subjected to validation with high-depth amplicon sequencing using custom Ampliseq panels on the same DNA on which WES was performed for all tumor and matched normal DNA from all six cases. Validation of putative WES somatic variants identified in the intratumor biopsies and technical replicates was performed separately. The validation panel for the intratumor biopsies included all putative mutations identified from at least one of the three pipelines (tumor-only, cohort-normal and matched-normal) described above. The validation panel for the technical replicates included putative mutations identified by the matched-normal pipeline only. Amplicons were successfully designed for 93.0% (1401/1508, range 91.0%–95.1% per patient) and 93.4% (741/793, range 85.5%–96.9% per patient) of the unique mutations for the biological replicates and the technical replicates, respectively. Putative mutations identified from WES for which amplicons could not be designed were excluded from further analyses.

Amplicon sequencing was performed to a median depth of 604x (range 363x–1519x) and 602x (range 460x–3102x) for the intratumor biopsies and technical replicates respectively (**Table S1**). Paired-end reads in FASTQ format were aligned to the reference human genome GRCh37 using the Torrent Mapping Alignment Program (TMAP, v3.4.1, https://github.com/iontorrent/TS/tree/master/Analysis/TMAP). Local realignment was performed using GATK (v3.1.1). Putative mutations were interrogated using pileup files generated using samtools mpileup (version 1.2 htslib 1.2.1)(Li, 2011), using reads with mapping quality of at least 1. For a given sample, mutations sequenced to ≤ 50x total depth were considered “low depth”, and mutations present at VAF ≤ 1% were considered “absent”. Mutations were considered “germline-like” if the VAF in the tumor was < 5 times than that of the VAF in the germline. The remaining mutations were considered validated to be “somatic”. These definitions are summarized in **Table S2**.

For each pair of technical replicates, variants validated to be somatic in both samples were considered “concordant”. Variants classified as low depth in either of the tumor samples were considered “low depth”. Variants that were validated to be absent in both samples were considered “absent”. Variants classified as germline-like in both tumor samples, or classified as germline-like in one sample and absent in the other sample, were considered “germline-like. Variants validated to be somatic in one of the two samples and germline or absent in the other sample were considered to be “discordant”. Similarly, for the multiple biopsies from the same cancer, variants validated as somatic in all three biopsies were considered “homogeneous”. Variants that were validated to be absent from all three biopsies were considered as “absent”. Variants classified as low depth in any of the three biopsies were considered as “low depth”. Variants that were validated to be germline-like in one of the biopsies and germline-like or absent in the other

two were considered “germline-like”. The remaining variants that were validated as somatic in 1 or 2 of the biopsies and germline or absent in the other biopsies were considered as “heterogeneous”. These definitions are summarized in **Table S2**.

Assessing technical variance and intra-tumor genetic heterogeneity (ITGH)

Concordance in putative somatic mutations as defined in the technical replicates by each of the three WES pipelines prior to validation was assessed using the Jaccard index (Levandowsky et al., 1971). The extent of discordance or technical noise involved in calling somatic mutations by a given pipeline was assessed by the Jaccard distance, which was defined as 1–Jaccard index, where 0 would indicate identical calls and 1 completely non-overlapping calls within each pair.

ITGH was assessed by comparing the somatic mutations identified in the three intratumor biopsies from the same tumor. The extent of ITGH for each case was then estimated by the Jaccard distance considering calls made on all three intratumor biopsies (1-homogeneous variants/union of somatic variants).

Identification of pathogenic and potentially pathogenic mutations

All validated non-synonymous mutations were classified as likely pathogenic or passenger mutations using *in silico* methods. For missense SNVs, their potential functional effects were assessed using a combination of MutationTaster (Schwarz et al., 2010) and CHASM (breast classifier) (Carter et al., 2009). SNVs defined as non-deleterious/passenger by both MutationTaster and CHASM were considered passenger mutations, as this combination was previously shown to have the highest negative predictive value (Martelotto et al., 2014). Non-passenger missense SNVs were defined as likely pathogenic if they were recurrent hotspot mutations (Chang et al., 2016), or predicted as “driver” or “cancer” alterations by CHASM or FATHMM (Shihab et al., 2013), respectively.

Pathogenicity of somatic in-frame indels was assessed using MutationTaster (Schwarz et al., 2010) and Protein Variation Effect Analyzer (PROVEAN) (Choi et al., 2012). In-frame indels predicted to be neutral by either were considered as passenger mutations, otherwise in-frame indels were considered likely pathogenic if they were associated with haploinsufficient affected genes (Dang et al., 2008), loss of the wild-type allele (based on FACETS (Shen and Seshan, 2016), see above), or at least one of the three cancer gene datasets (127 significant mutated genes (Kandoth et al., 2013a), the Cancer Gene Census (Futreal et al., 2004a) and Cancer5000-S gene set (Lawrence et al., 2014a)). Frameshift indels, splice donor/acceptor mutations and truncating mutations were considered potentially pathogenic if they were associated with loss of the wild-type allele (based on FACETS (Shen and Seshan, 2016)) or haploinsufficient affected genes (Dang et al., 2008), or affected cancer genes (Futreal et al., 2004b; Kandoth et al., 2013b; Lawrence et al., 2014b). Mutations that did not satisfy the above criteria were considered passenger mutations. Lists and characteristics of somatic variants detected by WES and Ampliseq on technical replicates are listed in **Table S3**. Somatic variants detected by WES and Ampliseq on the intratumor biopsies are listed in **Table S4**.

Identification of subclonal mutations

The clonality of putative somatic mutations identified from WES analysis was inferred using ABSOLUTE (Carter et al., 2012) using the segmented copy number log ratio from FACETS and the number of reads supporting the reference and the alternate alleles of the mutations obtained from WES as previously described (Ng et al., 2017a). The clonality of validated somatic mutations by Ampliseq was inferred using the number of reads supporting the reference and the alternate alleles of the mutations obtained by Ampliseq. A mutation was classified as clonal if its probability of being clonal was >50% or if the upper bound of the 95% confidence interval of its CCF was 100% (Landau et al., 2013).

Mutational signature analysis and mappability

We performed mutational signature analysis using the R package deconstructSigs (Rosenthal et al., 2016) as previously described (Ng et al., 2017b). First, the fraction of mutations found in each of the 96 possible trinucleotide contexts was calculated to build the mutational profile for each sample, normalized by the number of times each trinucleotide context is observed in the sequencing regions. For the pooled analysis, mutations from all samples were combined to result in a “pooled sample” profile. Next, the mutation profile was reconstructed with minimum error by

iteratively inferring the weighted contribution of each of the 30 reference signatures. Definition and interpretation of mutational signatures were obtained from COSMIC (http://cancer.sanger.ac.uk/cosmic/signatures).

We assessed mappability of SNVs in the GRCh37/hg19 reference genome using the 75bp CRG Alignability track available in the UCSC genome browser (http://genome.ucsc.edu/cgi-bin/hgTrackUi?db=hg19&g=wgEncodeMapability). The CRG Alignability track displays how uniquely 75-mer sequences align to different regions of the genome (Derrien et al., 2012). The mappability score is defined as the reciprocal of the number of matches found in the genome. A mappability score of 1 is considered high as it indicates a unique match.

To assess the effects of sequencing alignment using the newer BWA mem algorithm (http://bio-bwa.sourceforge.net/bwa.shtml) and of read length on variant identification in regions of low mappability, we obtained 10 tumor-normal pairs from The Cancer Genome Atlas breast cancer cohort (Kobolt et al., 2012a). These 10 tumor-normal pairs were sequenced using the 100bp paired-end sequencing. We 1) trimmed the raw sequences to 75bp and aligned the data with BWA aln (Li and Durbin, 2009), as we performed for our 6 cases in our manuscript, 2) trimmed the raw sequences to 75bp and aligned the data with BWA mem (http://bio-bwa.sourceforge.net/bwa.shtml), and 3) aligned the 100bp reads using BWA mem, then downsampled the resulting BAM files to 75% such that the overall sequencing depth largely matched the first 2 processing approaches. Mutation calling was performed using the matched-normal pipeline. Mappability was computed as described above.

Clonal inference

We performed clonal inference using PyClone (Roth et al., 2014), using the read counts from high-depth Ampliseq sequencing for all validated homogeneous and heterogeneous mutations. Major and minor copy numbers for each mutation, as well as tumor purity, were defined using FACETS (as described above). Case 2 biorep C, for which purity could not be estimated by FACETS, was set to 0.2. 10,000 iterations of Markov Chain Monte Carlo sampling were performed with the first 1,000 iterations discarded as "burn-in". Clusters composed of single mutation were discarded.

To assess the impact of sequencing depth on clonal inference, we performed a downsampling analysis. Specifically, we downsampled the number of reads for each mutation, at increments of 2.5% up to 25%, then at 5% up to 95%, of the original depth. Each downsampling experiment was performed 20 times. The downsampled number of reads was used as input to PyClone and clonal inference performed as described above. Clonal inference error was computed in relation to the clusters inferred from no downsampling. Clonal inference error was calculated as 1-(the number of mutations in the same clusters as no downsampling/the total number of mutations). All somatic mutations were included, regardless if they would have been considered somatic at the reduced depth.

SUPPLEMENTAL REFERENCES

Carter, H., Chen, S., Isik, L., Tyekucheva, S., Velculescu, V.E., Kinzler, K.W., Vogelstein, B., and Karchin, R. (2009). Cancer-specific high-throughput annotation of somatic mutations: computational prediction of driver missense mutations. Cancer Res. *69*, 6660–6667.

Carter, S.L., Cibulskis, K., Helman, E., McKenna, A., Shen, H., Zack, T., Laird, P.W., Onofrio, R.C., Winckler, W., Weir, B.A., et al. (2012). Absolute quantification of somatic DNA alterations in human cancer. Nat. Biotechnol. *30*, 413–421.

Chang, M.T., Asthana, S., Gao, S.P., Lee, B.H., Chapman, J.S., Kandoth, C., Gao, J., Socci, N.D., Solit, D.B., Olshen, A.B., et al. (2016). Identifying recurrent mutations in cancer reveals widespread lineage diversity and mutational specificity. Nat. Biotechnol. *34*, 155–163.

Choi, Y., Sims, G.E., Murphy, S., Miller, J.R., and Chan, A.P. (2012). Predicting the functional effect of amino acid substitutions and indels. PLoS One *7*, e46688.

Cibulskis, K., Lawrence, M.S., Carter, S.L., Sivachenko, A., Jaffe, D., Sougnez, C., Gabriel, S., Meyerson, M., Lander, E.S., and Getz, G. (2013). Sensitive detection of somatic point mutations in impure and heterogeneous cancer samples. Nat. Biotechnol. *31*, 213–219.

Cingolani, P., Platts, A., Wang, L.L., Coon, M., Nguyen, T., Wang, L., Land, S.J., Lu, X., and Ruden, D.M. (2012) A program for annotating and predicting the effects of single nucleotide polymorphisms, SnpEff. Fly, *6*, 80-92.

Dang, V.T., Kassahn, K.S., Marcos, A.E., and Ragan, M.A. (2008). Identification of human haploinsufficient genes and their genomic proximity to segmental duplications. Eur. J. Hum. Genet. *16*, 1350–1357.

De Mattos-Arruda, L., Weigelt, B., Cortes, J., Won, H.H., Ng, C.K.Y., Nuciforo, P., Bidard, F.-C., Aura, C., Saura, C., Peg, V., et al. (2014). Capturing intra-tumor genetic heterogeneity by de novo mutation profiling of circulating cell-free tumor DNA: a proof-of-principle. Ann. Oncol. *25*, 1729–1735.

Derrien, T., Estellé, J., Sola, S.M., Knowles, D.G., Raineri, E., Guigó, R., and Ribeca, P. (2012). Fast Computation and Applications of Genome Mappability. PLoS One *7*, e30377.

Futreal, P.A., Andrew Futreal, P., Lachlan, C., Mhairi, M., Thomas, D., Timothy, H., Richard, W., Nazneen, R., and Stratton, M.R. (2004a). A census of human cancer genes. Nat. Rev. Cancer *4*, 177–183.

Futreal, P.A., Andrew Futreal, P., Lachlan, C., Mhairi, M., Thomas, D., Timothy, H., Richard, W., Nazneen, R., and Stratton, M.R. (2004b). A census of human cancer genes. Nat. Rev. Cancer *4*, 177–183.

Kandoth, C., Cyriac, K., McLellan, M.D., Fabio, V., Kai, Y., Beifang, N., Charles, L., Mingchao, X., Qunyuan, Z., McMichael, J.F., et al. (2013a). Mutational landscape and significance across 12 major cancer types. Nature *502*, 333–339.

Kandoth, C., Cyriac, K., McLellan, M.D., Fabio, V., Kai, Y., Beifang, N., Charles, L., Mingchao, X., Qunyuan, Z., McMichael, J.F., et al. (2013b). Mutational landscape and significance across 12 major cancer types. Nature *502*, 333–339.

Koboldt, D.C., Fulton, R.S., McLellan, M.D., Schmidt, H., Kalicki-Veizer, J., McMichael, J.F., Fulton, L.L., Dooling, D.J., Ding, L., Mardis, E.R., et al. (2012a). Comprehensive molecular portraits of human breast tumours. Nature *490*, 61–70.

Koboldt, D.C., Zhang, Q., Larson, D.E., Shen, D., McLellan, M.D., Lin, L., Miller, C.A., Mardis, E.R., Ding, L., and Wilson, R.K. (2012b). VarScan 2: somatic mutation and copy number alteration discovery in cancer by exome sequencing. Genome Res. *22*, 568–576.

Landau, D.A., Carter, S.L., Stojanov, P., McKenna, A., Stevenson, K., Lawrence, M.S., Sougnez, C., Stewart, C., Sivachenko, A., Wang, L., et al. (2013). Evolution and impact of subclonal mutations in chronic lymphocytic leukemia. Cell *152*, 714–726.

Lawrence, M.S., Stojanov, P., Mermel, C.H., Robinson, J.T., Garraway, L.A., Golub, T.R., Meyerson, M., Gabriel, S.B., Lander, E.S., and Getz, G. (2014a). Discovery and saturation analysis of cancer genes across 21 tumour types. Nature *505*, 495–501.

Lawrence, M.S., Stojanov, P., Mermel, C.H., Robinson, J.T., Garraway, L.A., Golub, T.R., Meyerson, M., Gabriel, S.B., Lander, E.S., and Getz, G. (2014b). Discovery and saturation analysis of cancer genes across 21 tumour types. Nature *505*, 495–501.

Levandowsky, M., Michael, L., and David, W. (1971). Distance between Sets. Nature *234*, 34–35.

Li, H., and Durbin, R. (2009). Fast and accurate short read alignment with Burrows-Wheeler transform. Bioinformatics *25*, 1754–1760.

McKenna, A., Hanna, M., Banks, E., Sivachenko, A., Cibulskis, K., Kernytsky, A., Garimella, K., Altshuler, D., Gabriel, S., Daly, M., et al. (2010). The Genome Analysis Toolkit: a MapReduce framework for analyzing next-generation DNA sequencing data. Genome Res. *20*, 1297–1303.

Ng, C.K.Y., Bidard, F.-C., Piscuoglio, S., Geyer, F.C., Lim, R.S., de Bruijn, I., Shen, R., Pareja, F., Berman, S.H., Wang, L., et al. (2017a). Genetic Heterogeneity in Therapy-Naïve Synchronous Primary Breast Cancers and Their Metastases. Clin. Cancer Res. *23*, 4402–4415.

Ng, C.K.Y., Piscuoglio, S., Geyer, F.C., Burke, K.A., Pareja, F., Eberle, C., Lim, R., Natrajan, R., Riaz, N., Mariani, O., et al. (2017b). The Landscape of Somatic Genetic Alterations in Metaplastic Breast Carcinomas. Clin. Cancer Res. *23*, 3859–3870.

Qi, Y., Liu, X., Liu, C.-G., Wang, B., Hess, K.R., Symmans, W.F., Shi, W., and Pusztai, L. (2015). Reproducibility of Variant Calls in Replicate Next Generation Sequencing Experiments. PLoS One *10*, e0119230.

Robinson, J.T., Helga, T., Wendy, W., Mitchell, G., Lander, E.S., Gad, G., and Mesirov, J.P. (2011). Integrative genomics viewer. Nat. Biotechnol. *29*, 24–26.

Rosenthal, R., McGranahan, N., Herrero, J., Taylor, B.S., and Swanton, C. (2016). DeconstructSigs: delineating mutational processes in single tumors distinguishes DNA repair deficiencies and patterns of carcinoma evolution. Genome Biol. *17*, 31.

Roth, A., Khattra, J., Yap, D., Wan, A., Laks, E., Biele, J., Ha, G., Aparicio, S., Bouchard-Cote, A., Shah, S.P. (2014). PyClone: statistical inference of clonal population structure in cancer. Nat. Methods *11*, 396-398.

Saunders, C.T., Wong, W.S.W., Swamy, S., Becq, J., Murray, L.J., and Cheetham, R.K. (2012). Strelka: accurate somatic small-variant calling from sequenced tumor-normal sample pairs. Bioinformatics *28*, 1811–1817.

Schwarz, J.M., Christian, R., Markus, S., and Dominik, S. (2010). MutationTaster evaluates disease-causing potential of sequence alterations. Nat. Methods *7*, 575–576.

Shen, R., and Seshan, V.E. (2016). FACETS: allele-specific copy number and clonal heterogeneity analysis tool for high-throughput DNA sequencing. Nucleic Acids Res. *44*, e131.

Shihab, H.A., Gough, J., Cooper, D.N., Stenson, P.D., Barker, G.L.A., Edwards, K.J., Day, I.N.M., and Gaunt, T.R. (2013). Predicting the functional, molecular, and phenotypic consequences of amino acid substitutions using hidden Markov models. Hum. Mutat. *34*, 57–65.

Yang, H., and Wang, K. (2015). Genomic variant annotation and prioritization with ANNOVAR and wANNOVAR. Nat. Protoc. *10*, 1556–1566.

Zook, J.M., Brad, C., Jason, W., David, M., Oliver, H., Winston, H., and Marc, S. (2014). Integrating human sequence data sets provides a resource of benchmark SNP and indel genotype calls. Nat. Biotechnol. *32*, 246–251.
